# Causal relationships between gut microbiota and depression/anxiety disorders: A 2-sample Mendelian randomization study

**DOI:** 10.1097/MD.0000000000039543

**Published:** 2024-09-06

**Authors:** Tianyue Fan, Lingxiao Li, Yi Chen

**Affiliations:** a Southwest Medical University, Luzhou, Sichuan, China; b School of Humanities and Management Science, Southwest Medical University, Luzhou, Sichuan, China.

**Keywords:** anxiety disorder, depression, gut microbiota, Mendelian randomization

## Abstract

Evidence shows that the composition of the gut microbiota (GM) is associated with depression and anxiety disorders. However, the causal relationship between them remains controversial. To investigate the potential causal relationship between the GM and depression/anxiety disorders and to identify specific bacterial taxa, we conducted a 2-sample Mendelian randomization (MR) analysis on the gut microbiome implicated in depression and anxiety disorders. We incorporated summary data from genome-wide association studies (GWAS) of the microbiome derived from 7738 individuals in the Dutch Microbiome Project and 18,340 individuals in the MiBioGen consortium as our exposure variable. Concurrently, the GWAS of depression and anxiety disorders was employed as our outcome variable. The principal estimates were procured using the inverse-variance weighted test complemented by 4 robust methods: MR Egger, weighted median, simple mode, and weighted mode. In addition, we performed comprehensive sensitivity and directionality analyses. The results showed that 5 bacterial taxa were positively correlated with depression, 6 were negatively correlated; 5 were positively correlated with anxiety disorders, and 11 were negatively correlated. This study provides new insights into the connection between the GM and the pathogenesis of depression and anxiety disorders and offers new perspectives for the diagnosis and treatment of these disorders.

## 1. Introduction

Depression and anxiety disorders are 2 of the most common mental disorders. Depression refers to a significant and persistent low mood, loss of pleasure or interest in things experienced by the patient.^[1]^ Approximately 3.8% of the world population suffers from depression, leading to more than 700,000 suicides each year.^[2]^ Anxiety disorders refer to intense, excessive, and persistent fear and worry that occur in everyday situations and are often accompanied by autonomic nervous symptoms and motor restlessness, which interfere with the patient’s normal functioning.^[3]^ Approximately 20% to 30% of people between the ages of 15 and 25 have experienced anxiety disorders, and some of them continue to persist.^[4,5]^ In addition, the COVID-19 pandemic has significantly increased the global burden of depression and anxiety disorders.^[6]^ Risk factors for depression and anxiety disorders include genetics, the environment, and epigenetics, with the role of multiple environmental factors in these disorders gaining more attention.^[1,3]^ Currently, effective treatments for these diseases include evidence-based psychotherapies and drug treatments (SSRIs/SNRIs, etc).^[1,7,8]^ However, existing treatment plans cannot meet the large and increasing disease burden, and patients urgently need additional treatment options to provide opportunities for disease relief.

The gut microbiota (GM) refers to the total microbial community in the human gastrointestinal tract. Increasing evidence suggests that the GM is not an isolated unit but communicates and influences the body’s major systems through various mechanisms.^[9]^ The gut–brain axis is a bidirectional communication system between the central nervous system and the gastrointestinal tract.^[10]^ Several observational or experimental studies have suggested that GM seems to have an effect on the central nervous system and neurological and mental diseases (including anxiety disorders, depression, Parkinson disease, etc) through interactions with the gut-brain axis, and the composition of the GM in these patients is significantly different from that in healthy people.^[11–14]^ According to Dinan et al, the GM may interact with neurological, humoral and immunological components of the body in the gastrointestinal tract, thereby affecting the function and development of the central nervous system.^[10]^ The specific pathways involved may include the production of various bioactive substances (neurotransmitters, short-chain fatty acids, acetates, and propionates), the induction of inflammatory cytokines, and even the triggering of the vagus nerve.^[10,15]^ Further studies have confirmed that modifying the composition of the GM through the administration of specific microbial agents or antibiotics can significantly affect psychobehavioral characteristics or susceptibility to neuropsychiatric disorders in patients and mice.^[11,14,16–19]^ Therefore, the GM seems to be another important target for the diagnosis and treatment of such neuropsychiatric diseases.^[20,21]^ However, the results of observational or experimental studies are susceptible to confounding factors and thus carry the risk of confounding relevance and causality. In addition, due to limitations in data volume and experimental ethics, these results often face the pitfalls of incompleteness and lack of clarity. Clarification of the independent causal relationship between specific gut bacteria and outcomes is considered necessary. Despite growing evidence that changes in the abundance of specific GM (e.g., Firmicutes, Actinobacteria, Bacteroidetes, etc) are associated with anxiety and depression, the causal relationship between the 2 is still uncertain.^[22–25]^

Mendelian randomization (MR) is a statistical method in which Single Nucleotide Polymorphisms (SNPs) are used to detect the causal effects of exposure factors on outcomes.^[26]^ This method uses genetic variations determined before birth as instrumental variables to evaluate causal effects, effectively reducing the bias caused by confounding factors or reverse causality and thereby facilitating causal inference.

In this study, to explore the potential causal relationship between the GM and depression/anxiety disorders and to determine specific bacterial taxa, we conducted an MR study based on genome-wide association studies (GWAS) data.

## 2. Materials and methods

### 2.1. Data sources

The depression data were obtained from the GWAS Catalog of EMBL-EBI.^[27]^ This dataset includes 13,559 cases and 435,855 controls, with 24,184,163 SNPs. The anxiety disorder data were obtained from a dataset created by the Neale lab in 2018 as part of the UK Biobank project.^[28]^ This dataset includes 1092 cases and 360,102 controls, with 9440,635 SNPs. The human gut microbiome data we used were obtained from GWASs of Dutch Microbiome Project with 7738 individuals and MiBioGen consortium with 18,340 individuals.^[29,30]^ All the data are publicly available.

### 2.2. Instrumental variable selection

Our research analyzed the exposure factors at 6 levels, phylum, class, order, family, genus, and species, and selected suitable instrumental variables through a series of quality control steps.

**Figure 1.**
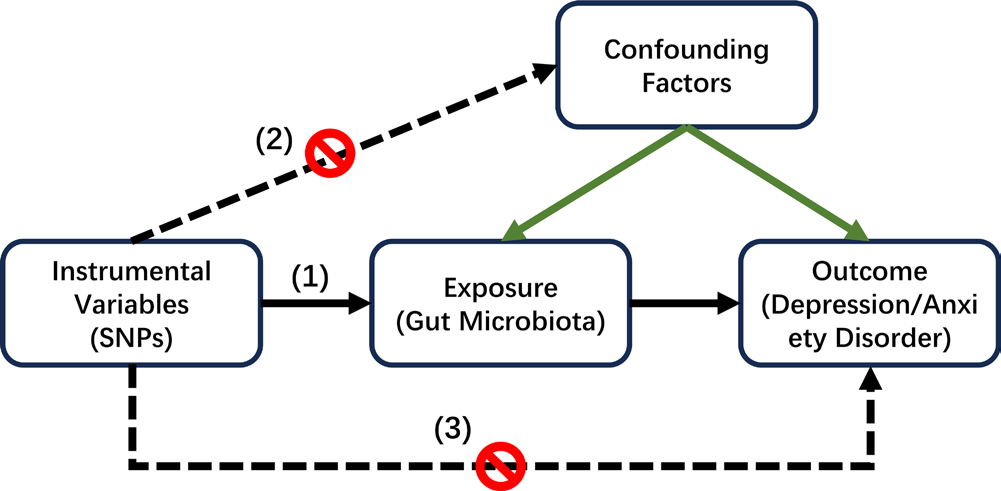
Three assumptions for Mendelian randomization. (1) Relevance assumption, (2) independence assumption, and (3) exclusion restriction assumption.

The 3 major assumptions of MR (Fig. 1) are as follows: There is a strong correlation between the instrumental variables (SNPs) and the exposure factors (GM). It is required that each candidate IV is included in *P* < 5 × 10^−8^ to ensure the reliability of the results. The linkage disequilibrium (LD) threshold was set to *R*^2^ <0.001 and clumping distance = 10,000 kb to reduce the bias caused by LD. Genetic variation is unrelated to confounding factors. Genetic variation is unrelated to outcome variables.^[26,31]^

PhenoScanner^[32]^ was used to exclude confounding factors related to depression and anxiety disorders (smoking, alcohol, BMI, diet and educational attainment) and SNPs related to these 2 outcomes. These confounding factors are considered as common factors that may affect the outcome variables.^[33]^ SNPs with palindromic structures were automatically excluded to avoid distortion of strand orientation or allele coding. To eliminate the bias caused by weak instrumental variables, we calculated the *F* value for each SNP. SNPs with *F* values < 10 were considered weak instrumental variables.^[34]^

### 2.3. MR analysis

We conducted MR analysis on the GM with 2 outcomes (depression and anxiety disorders) respectively. The inverse-variance weighted (IVW) test was used as the main method and was supplemented by 4 methods: MR Egger, weighted median, simple mode, and weighted mode.^[26]^

### 2.4. Sensitivity analyses

The sensitivity analysis included a heterogeneity test, horizontal pleiotropy test, and leave-one-out analysis. The heterogeneity test used Cochran Q to determine the heterogeneity of SNPs.^[35]^ If the Cochran Q test is statistically significant (*P* ≤ .05), it indicates that the results have significant heterogeneity. The horizontal pleiotropy test used MR Egger for analysis.^[36]^ If there is a significant intercept (*P* > .05), it indicates that the study has horizontal pleiotropy. Leave-one-out analysis was used to determine whether the significant results were determined by a single SNP.^[37]^

### 2.5. Additional analyses

**Figure 2.**
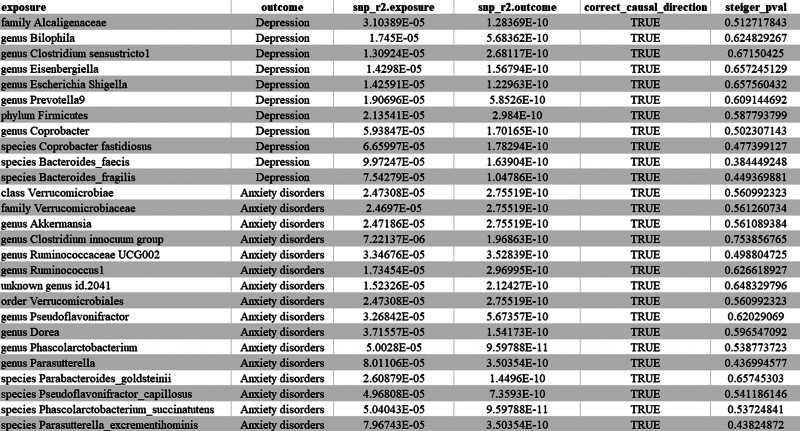
Steiger test results of depression/anxiety disorder.

We conducted a Steiger test to verify the directionality of the exposure and outcome and excluded possible reverse-positive results (Fig. 2; Additional Files 1 and 2, Supplemental Digital Content, http://links.lww.com/MD/N490).^[37]^

### 2.6. Statistical software and version

All the statistical analyses were performed using R version 4.3.2. MR analyses were performed using TwoSampleMR version 0.5.8.

## 3. Results

### 3.1. Major MR results for the GM associated with depression

**Figure 3.**
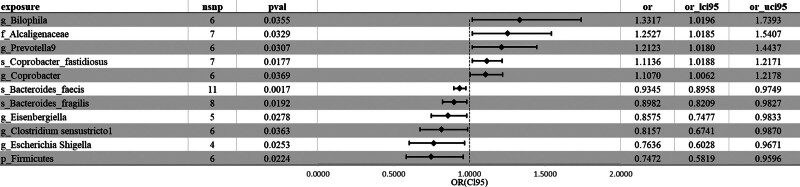
Major Mendelian randomization Results for the gut microbiota associated with depression.

MR analysis was performed on 412 types of bacteria at 6 levels, phylum, class, order, family, genus, and species, and 11 bacterial taxa related to depression were ultimately screened out (Additional File 3, Supplemental Digital Content, http://links.lww.com/MD/N490). All the selected SNPs had a *F* value > 10, which can be used to effectively avoid bias caused by weak instrumental variables. Among these taxa (Fig. 3; Figure S1, Supplemental Digital Content, http://links.lww.com/MD/N486), the bacterial taxa positively correlated with depression include species *Coprobacter fastidiosus* (OR = 1.114, 95% CI: 1.019, 1.217, *P* = .018, β = 0.108), genus *Prevotella9* (OR = 1.212, 95% CI: 1.018, 1.444, *P* = .031, β = 0.193), family Alcaligenaceae (OR = 1.253, 95% CI: 1.018, 1.541, *P* = .033, β = 0.225), genus *Bilophila* (OR = 1.332, 95% CI: 1.020, 1.739, *P* = .036, β = 0.286), genus *Coprobacter* (OR = 1.107, 95% CI: 1.006, 1.218, *P* = .037, β = 0.102). The bacterial taxa negatively correlated with depression include: species *Bacteroides faecis* (OR = 0.935, 95% CI: 0.896, 0.975, *P* = .002, β = −0.068), species *Bacteroides fragilis* (OR = 0.898, 95% CI: 0.821, 0.983, *P* = .019, β = −0.107), phylum Firmicutes (OR = 0.747, 95% CI: 0.582, 0.960, *P* = .022, β = −0.291), genus *Escherichia Shigella* (OR = 0.764, 95% CI: 0.603, 0.967, *P* = .025, β = −0.270), genus *Eisenbergiella* (OR = 0.857, 95% CI: 0.748, 0.983, *P* = .028, β = −0.154), genus *Clostridium sensustricto1* (OR = 0.816, 95% CI: 0.674, 0.987, *P* = .036, β = −0.204). The MR-Egger intercept test did not reveal significant pleiotropy (*P* > .05), indicating that our instrumental variables met the exclusion restriction assumption. Heterogeneity tests also did not reveal significant heterogeneity (*P* > .05), indicating that our instrumental variables had a consistent effect direction. Leave-one-out sensitivity analysis also did not reveal any outliers, indicating that our results are robust.

### 3.2. Major MR results for the GM associated with anxiety disorders

**Figure 4.**
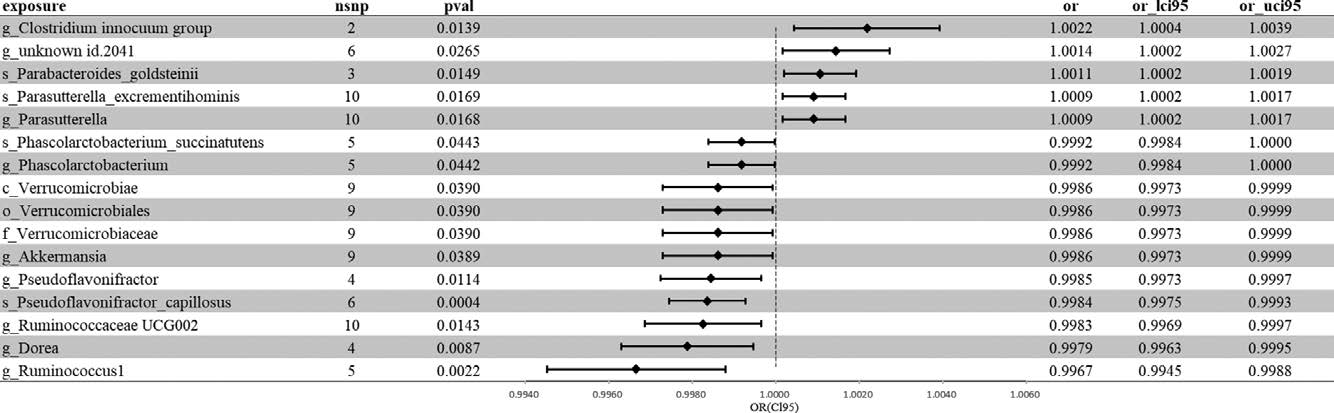
Major Mendelian randomization Results for the gut microbiota associated with anxiety disorder.

We screened out 16 gut bacterial taxa related to anxiety disorders (Additional File 4, Supplemental Digital Content, http://links.lww.com/MD/N490). All the selected SNPs had a *F* value > 10, which can be used to effectively avoid bias caused by weak instrumental variables. Among the remaining 16 taxa (Fig. 4; Figure S2, Supplemental Digital Content, http://links.lww.com/MD/N487), the bacterial taxa positively correlated with anxiety disorders include: genus *Clostridium innocuum group* (OR = 1.002, 95% CI: 1.000, 1.004, *P* = .014, β = 0.002), species *Parabacteroides goldsteinii* (OR = 1.001, 95% CI: 1.000, 1.002, *P* = .015, β = 0.001), genus *Parasutterella* (OR = 1.001, 95% CI: 1.000, 1.002, *P* = .017, β = 0.001), species *Parasutterella excrementihominis* (OR = 1.001, 95% CI: 1.000, 1.002, *P* = .017, β = 0.001), unknown genus (id.2041) (OR = 1.001, 95% CI: 1.000, 1.003, *P* = .026, β = 0.001). the bacterial taxa negatively correlated with anxiety disorders include: species *Pseudoflavonifractor capillosus* (OR = 0.998, 95% CI: 0.997, 0.999, *P* = .000, β = −0.002), genus *Ruminococcus1* (OR = 0.997, 95% CI: 0.995, 0.999, *P* = .002, β = −0.003), genus *Dorea* (OR = 0.998, 95% CI: 0.996, 0.999, *P* = .009, β = −0.002), genus *Pseudoflavonifractor* (OR = 0.998, 95% CI: 0.997, 1.000, *P* = .011, β = −0.002), genus *Ruminococcaceae UCG002* (OR = 0.998, 95% CI: 0.997, 1.000, *P* = .014, β = −0.002), order Verrucomicrobiales (OR = 0.999, 95% CI: 0.997, 1.000, *P* = .039, β = −0.001), genus *Akkermansia* (OR = 0.999, 95% CI: 0.997, 1.000, *P* = .039, β = −0.001), family Verrucomicrobiaceae (OR = 0.999, 95% CI: 0.997, 1.000, *P* = .039, β = −0.001), class Verrucomicrobiae (OR = 0.999, 95% CI: 0.997, 1.000, *P* = .039, β = −0.001), genus *Phascolarctobacterium* (OR = 0.999, 95% CI: 0.998, 1.000, *P* = .044, β = −0.001), species *Phascolarctobacterium succinatutens* (OR = 0.999, 95% CI: 0.998, 1.000, *P* = .044, β = −0.001). Our MR-Egger intercept test did not reveal significant pleiotropy (*P* > .05), indicating that our instrumental variables met the exclusion restriction assumption. Moreover, our heterogeneity test did not reveal significant heterogeneity (*P* > .05), indicating that our instrumental variables had a consistent effect direction. Our leave-one-out sensitivity analysis also did not reveal any outliers, indicating that our results are robust.

### 3.3. Sensitivity analyses

The heterogeneity of SNPs was determined using Cochran Q, and the Q_pval of both MR Egger and IVW were >.05, indicating no heterogeneity (Additional Files 5 and 6, Supplemental Digital Content, http://links.lww.com/MD/N490). The horizontal pleiotropy between the instrumental variables and the outcomes was analyzed using MR Egger, and all *P* values were >.05, providing no evidence of horizontal pleiotropy (Additional Files 7 and 8, Supplemental Digital Content, http://links.lww.com/MD/N490). Leave-one-out analysis revealed that no single SNP dominated the overall effect (Figure S3, Supplemental Digital Content, http://links.lww.com/MD/N488 and Figure S4, Supplemental Digital Content, http://links.lww.com/MD/N489).

## 4. Discussion

This study used data from a large cohort study in Europe, separately analyzed the 16S rRNA gene sequencing of the GM and depression/anxiety disorders, and provided evidence for the causal relationship between the GM and depression and anxiety disorders. The results showed that there was a significant causal relationship between the GM and depression, and some bacterial taxa were positively correlated with depression, while others were negatively correlated. These bacterial taxa may be potential biomarkers or therapeutic targets for depression. The 5 bacterial taxa that have the most significant impact on depression are as follows: The genus *Bilophila* comprises anaerobic bacteria that are mainly distributed in the human colon and appendix and can use bile acids as energy to produce hydrogen sulfide and propionic acid. The *Bilophila* genus of bacteria has been found to be enriched in the intestines of patients with major depressive disorder (MDD).^[38]^ The phylum Firmicutes comprises gram-positive bacteria that are widely present in the human gut and are involved in many important metabolic processes, such as the synthesis of short-chain fatty acids, protein degradation, and vitamin synthesis. The Firmicutes phylum of bacteria is closely related to gut health and immune system balance. Research has shown that, unlike patients with bipolar depression, healthy patients have greater enrichment of the phylum Firmicutes.^[24]^ The genus *Escherichia Shigella* comprises gram-negative bacteria that are present mainly in the human large intestine. The *Escherichia Shigella* genus of bacteria is related to intestinal inflammation, diarrhea, and food poisoning, but a higher abundance of *Escherichia Shigella* has been found to be associated with a lower risk of depressive symptoms.^[39]^ The family Alcaligenaceae is a bacterial family of the order Pseudomonadales that is composed mainly of aerobic or facultative anaerobic gram-negative bacilli. Alcaligenaceae are related to GM imbalance and metabolic disorders and may induce depression by affecting the gut–liver–brain axis.^[40]^ The genus *Prevotella9* is a bacterial genus of the Pseudomonadales order and is composed mainly of anaerobic gram-negative bacilli. According to our results, bacteria of the *Prevotella9* genus are positively correlated with depression. However, several observations researches show that the abundance of the family Prevotellaceae is low in MDD patients while others not.^[20,41,42]^ This suggests that the role of bacteria may not be directly related to the abundance they present in disease. Other possible reasons include different inclusion criteria for study subjects, different bacterial stratification, and different testing standards.

In addition, we also found a causal relationship between several gut bacterial taxa and anxiety disorders in our study, but the relationship was not significant. Different from depression, the effect of GM on anxiety disorders is slight, suggesting that GM may not play much of a role in the initiation and development of anxiety disorders independently. However, this does not mean that these bacteria are not communicating with the disease. Whether they will play a special role in complex clinical situations needs to be examined in subsequent clinical practice. These bacteria might also serve as potential biomarkers or therapeutic targets for anxiety disorders. The 3 bacterial taxa that have the most significant impact on anxiety disorders are as follows: The genus *Clostridium innocuum* is a group of anaerobic bacteria from the phylum Firmicutes that is positively correlated with anxiety disorders. These bacteria can produce neuroactive substances, such as gamma-aminobutyric acid (GABA) and acetylcholine (ACh) in the gut.^[25]^ These substances may increase anxiety by affecting the balance of neurotransmitters in the brain. The species *Pseudoflavonifractor capillosus* is an anaerobic bacterium of the order Clostridiales that is negatively correlated with anxiety disorders. The genus *Ruminococcus1* is a group of anaerobic bacteria from the order Clostridiales that is negatively correlated with anxiety disorders. It can ferment various carbohydrates in the gut to produce short-chain fatty acids. These substances may improve mood and cognitive function by regulating neurotransmitters in the brain, thereby reducing the risk of anxiety.^[43]^

More observational studies support the evidence of association between GM and depression/anxiety disorders. Researchers from Peking University found higher proportions of *Bacteroides* and *Prevotella* in depression.^[41]^ This is consistent with the elevated *Prevotella* found by Zhu et al in patients with anxiety and depression, while healthy populations contained more *Faecalibacterium*.^[42]^ Zhou et al found more *Escherichia Shigella* and *Flavonifractor* in pediatric depression.^[44]^ Systematic reviews and meta-analyses provided more comprehensive evidence. Changes in abundance occurring in the phylum Firmicutes, Bacteroidetes, and Proteobacteria were generally described.^[20,39,45–47]^

Depression and anxiety often present as comorbidities but are still different entities. The commonality between anxiety and depression includes negative affect (general psychological distress or negative emotions), while the unique feature of anxiety is physiological hyperarousal (tension, trembling), and the unique aspect of depression is low positive affect/lack of joy (loss of interest, despair).^[48]^ These unique symptoms of anxiety and depression persist across all age groups.^[49]^ This study discussed the relationship between the GM and these 2 mental disorders separately. Future research can increase the analysis of the GM in comorbid patients to avoid interference between outcome variables. In addition, whether the microbiota that appears simultaneously in patients with depression or anxiety disorders plays a more important role in the onset of comorbid patients is worth exploring.

This study has several advantages. We used the latest GWAS data of 418 bacterial taxa for MR research, while the data used in previous studies may be insufficient and outdated. Microbial taxonomic information at the species level was introduced for the first time. In addition, there is no reliable experimental or observational research support for the causal relationship between some microbiota and outcomes; therefore, this study can provide reliable evidence and ideas for further research in the future. Finally, multiple methods were employed to assess the sensitivity of the associations and to validate the robustness of the results.

This study has certain limitations. First, we did not refine the types of depression/anxiety disorders but analyzed a category of diseases as a whole. This approach may overlook the heterogeneity between different subtypes. Future research should consider a more detailed classification of the outcome to explore a more precise causal relationship. Additionally, The GWAS data used in our study were all from European populations, which can make the results possibly unsuitable for generalization to other ethnicities or populations. Third, this study did not conduct reverse MR; that is, depression/anxiety disorders were used as exposure factors to assess their potential impact on the GM. Reverse MR is able to visualize the magnitude of reverse causality, thus excluding possible biased forward results. To fill this gap, we introduced the Steiger test to assess the magnitude of reverse causality. It helps us to ensure that the selected SNPs have a greater correlation with exposure than with outcome, thus avoiding possible reverse causality. Although this method cannot directly detect reverse causality, it can still reduce the bias due to reverse causality to some extent. That is because only those results that passed the validation of the Steiger test were retained.

In conclusion, we comprehensively evaluated the potential causal relationship between the GM and depression/anxiety disorders. The microbiota related to depression included 5 positively correlated and 6 negatively correlated bacterial taxa. The microbiota related to anxiety disorders included 5 positively correlated and 11 negatively correlated bacterial taxa. A growing body of research has attempted to explain the impact of the GM on anxiety disorders and depression in terms of mechanisms. Li et al found that 3β-hydroxysteroid dehydrogenase (3β-HSD)-producing *Mycobacterium neoaurum* could induce depression in rats by degrading testosterone to androstenedione, while 3β-HSD was found to be enriched in men with depression.^[50]^ De Santa et al found that administration of a multi-strain probiotic mixture to mice could reverse symptoms of anxiety and depression in adult mice induced by postnatal maternal separation.^[51]^ *Roseburia* was found to be able to treat adolescent depression by balancing Trp-derived neurotransmitter metabolism and improving synaptogenesis and glial maintenance.^[52]^ As we mentioned earlier, neurotransmitters and SCFAs are important mediators of signaling from GM to the central nervous system. Butyrate, a SCFAs that can be produced by *Faecalibacterium* and *Coprococcus*, reduces intestinal inflammation by strengthening the epithelial defense barrier.^[53,54]^ Both of the bacteria are associated with higher quality of life and have been found to be depleted in depression.^[22,55]^ Some gut bacteria can produce GABA, which acts as an inhibitory neurotransmitter that can lead to manifestations of anxiety and depression in patients.^[55]^ Follow-up studies need to further explore how these bacteria with opposite effects differ in the underlying mechanisms affecting the disease. It is worth noting that these bacteria may not act as independent influencing factors in patients. Changes in the abundance of multiple bacteria in the patient gut occur at the same time. Are the effects resulting from these changes superimposed? Do different bacteria promote each other? Latest evidence that may be able to answer this question has been discovered. Zhao et al found that *Roseburia intestinalis* was able to ameliorate depressive symptoms by increasing homovanillic acid (HVA) production through increasing the abundance of *Bifidobacterium longum* in the intestine.^[56]^ More evidence needs to be obtained in subsequent studies or clinical practice to answer these questions. Our study may provide new insights into the connection between the GM and the pathogenesis of depression/anxiety disorders and offer new perspectives for the clinical diagnosis and treatment of patients. Comprehensive intervention in the GM of patients may improve patient symptoms and provide a good prognosis.

## Author contributions

**Data curation:** Tianyue Fan, Lingxiao Li.

**Writing – original draft:** Tianyue Fan.

**Writing – review & editing:** Tianyue Fan, Yi Chen.

**Project administration:** Yi Chen.

**Supervision:** Yi Chen.

**Validation:** Yi Chen.

## Supplementary Material


