# Supplementary material for: Causal relationships between gut microbiota and depression/anxiety disorders: A 2-sample Mendelian randomization study
Source: Medicine (Baltimore). 2024 Sep 6;103(36):e39543. doi: 10.1097/MD.0000000000039543 (PMC12431749; doi:10.1097/MD.0000000000039543)
Supplement: Supplementary file 1 [file medi-103-e39543-s001.pdf]

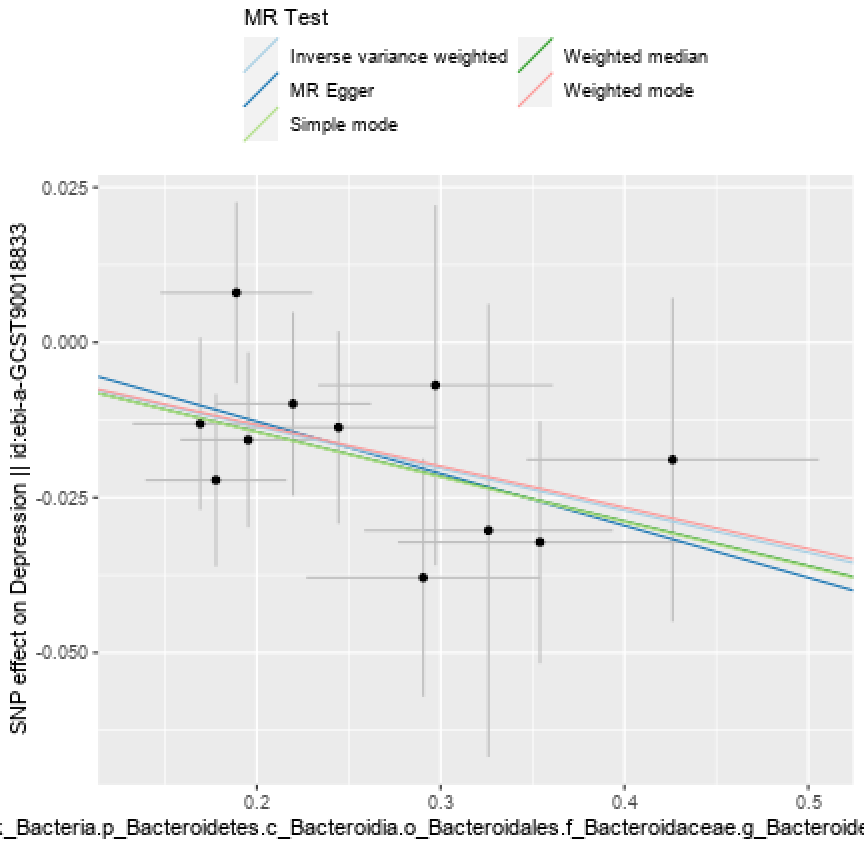

MR Test
Inverse variance weighted
MR Egger
Simple mode
Weighted median
Weighted mode
0.025
0.000
-0.025
-0.050
SNP effect on Depression || id:ebi-a-GCST90018833
0.2
0.3
0.4
0.5
_Bacteria.p_Bacteroidetes.c_Bacteroidia.o_Bacteroidales.f_Bacteroidaceae.g_Bacteroide

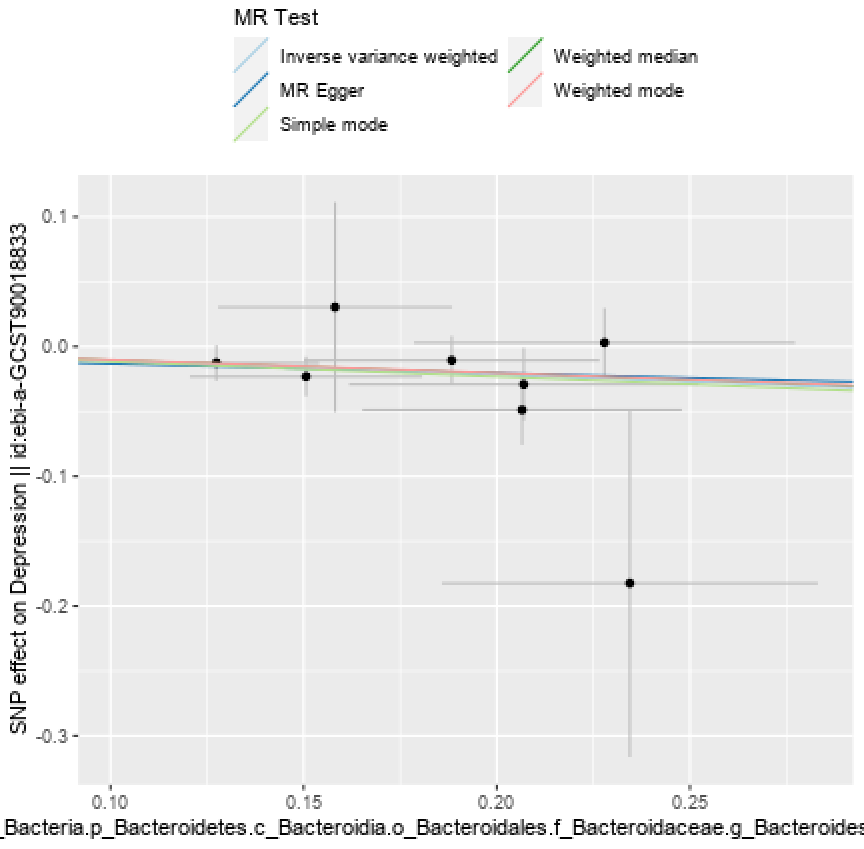

MR Test
Inverse variance weighted
MR Egger
Simple mode
Weighted median
Weighted mode
SNP effect on Depression || id:ebi-a-GCST90018833
0.1
0.0
-0.1
-0.2
-0.3
0.10
0.15
0.20
0.25
_Bacteria.p_Bacteroidetes.c_Bacteroidia.o_Bacteroidales.f_Bacteroidaceae.g_Bacteroides

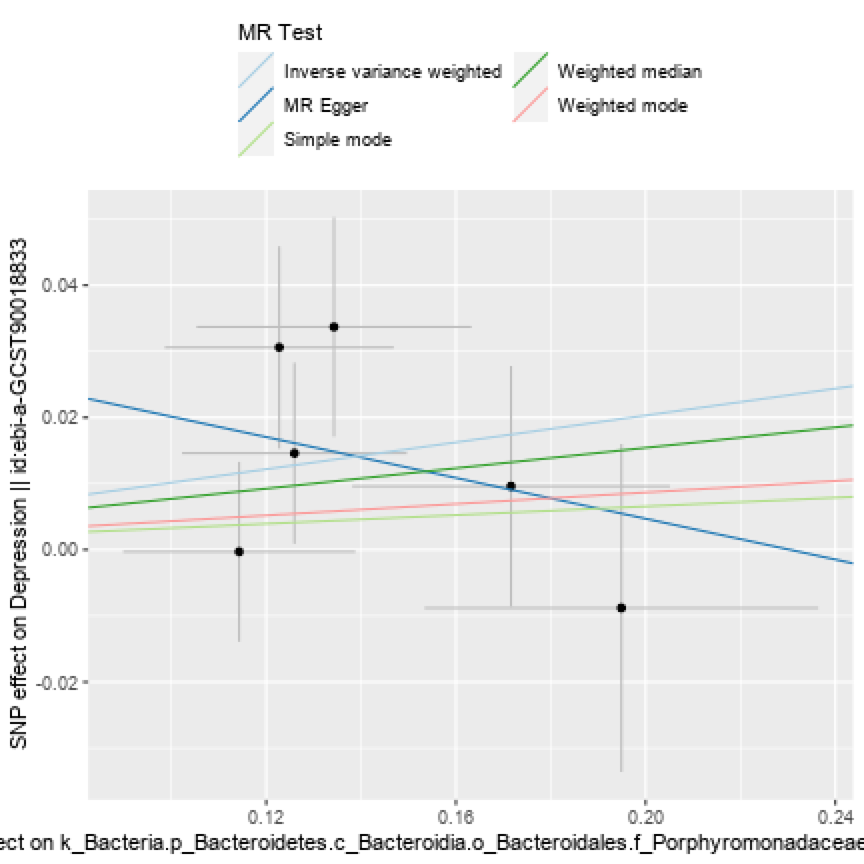

MR Test
Inverse variance weighted
MR Egger
Simple mode
Weighted median
Weighted mode
SNP effect on Depression || id:ebi-a-GCST90018833
0.04
0.02
0.00
-0.02
0.12
0.16
0.20
0.24
ect on k_Bacteria.p_Bacteroidetes.c_Bacteroidia.o_Bacteroidales.f_Porphyromonadaceae

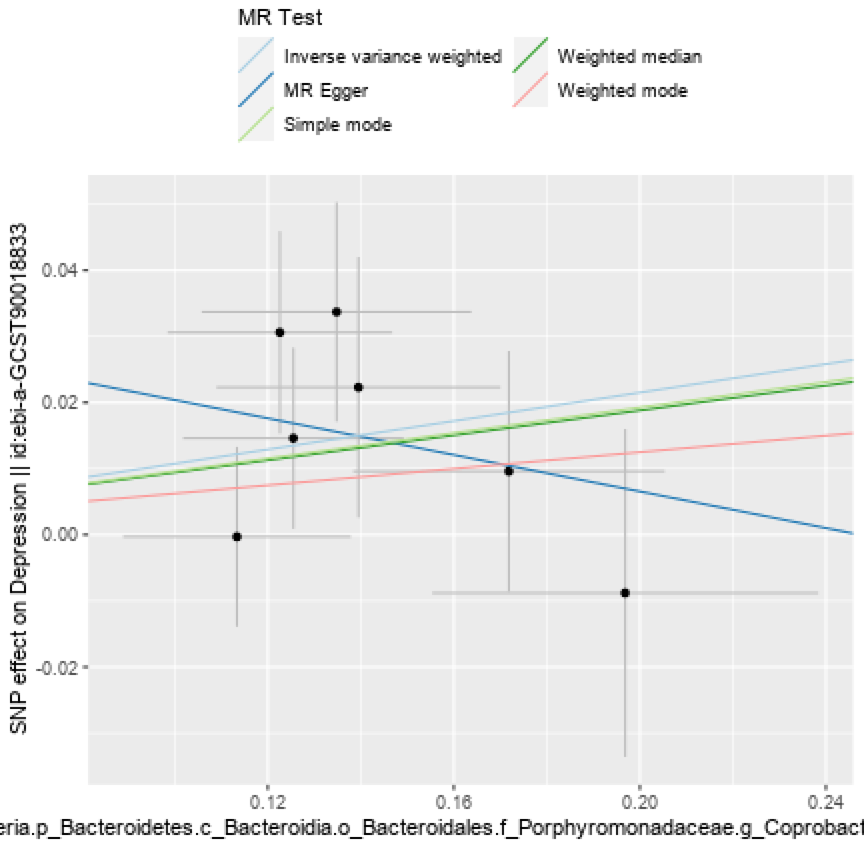
MR Test
Inverse variance weighted
MR Egger
Simple mode
Weighted median
Weighted mode
SNP effect on Depression || id:ebi-a-GCST90018833
0.04
0.02
0.00
-0.02
0.12
0.16
0.20
0.24
eria.p_Bacteroidetes.c_Bacteroidia.o_Bacteroidales.f_Porphyromonadaceae.g_Coprobact

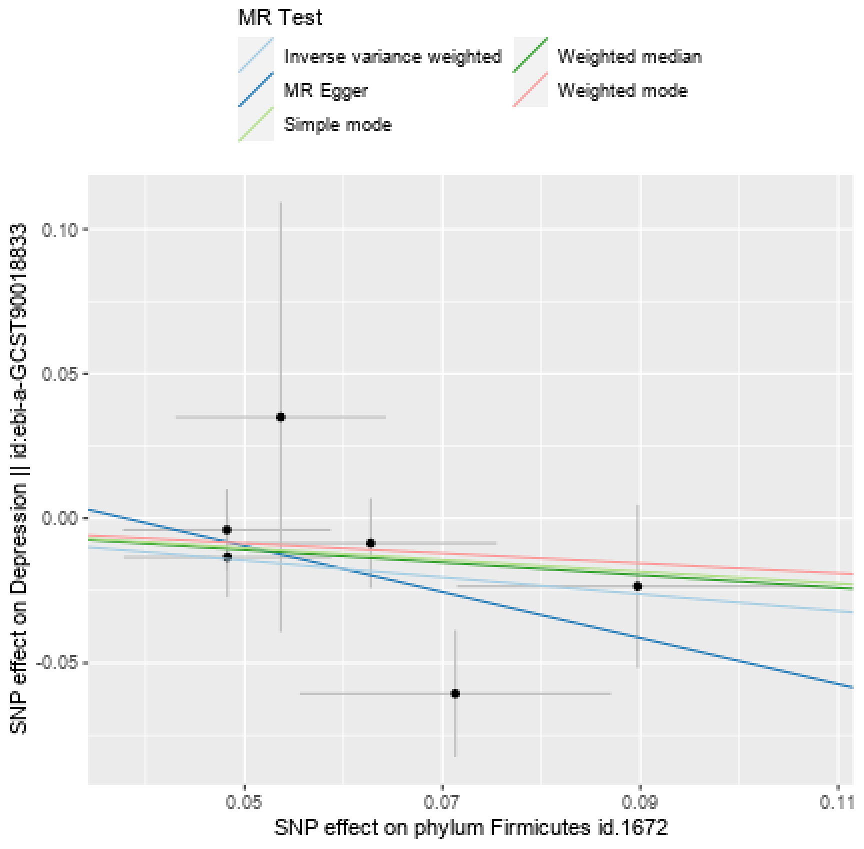
MR Test
Inverse variance weighted
MR Egger
Simple mode
Weighted median
Weighted mode
0.10
0.05
0.00
-0.05
SNP effect on Depression || id:ebi-a-GCST90018833
0.05
0.07
0.09
0.11
SNP effect on phylum Firmicutes id.1672

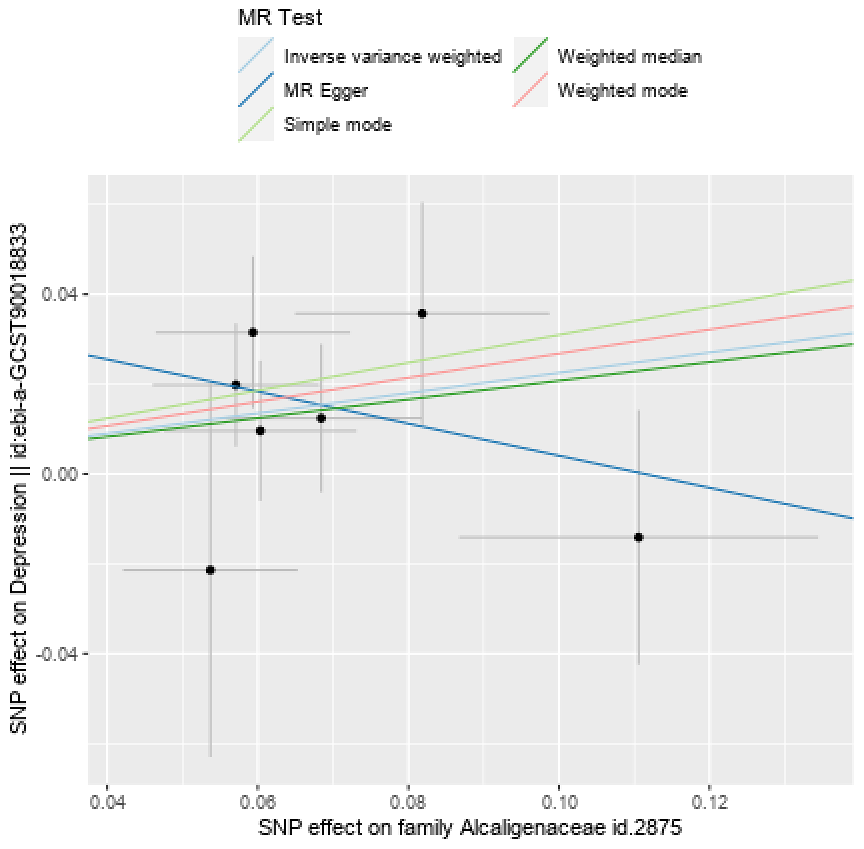

MR Test
Inverse variance weighted
MR Egger
Simple mode
Weighted median
Weighted mode
SNP effect on Depression || id:ebi-a-GCST90018833
0.04
0.00
-0.04
0.04
0.06
0.08
0.10
0.12
SNP effect on family Alcaligenaceae id.2875

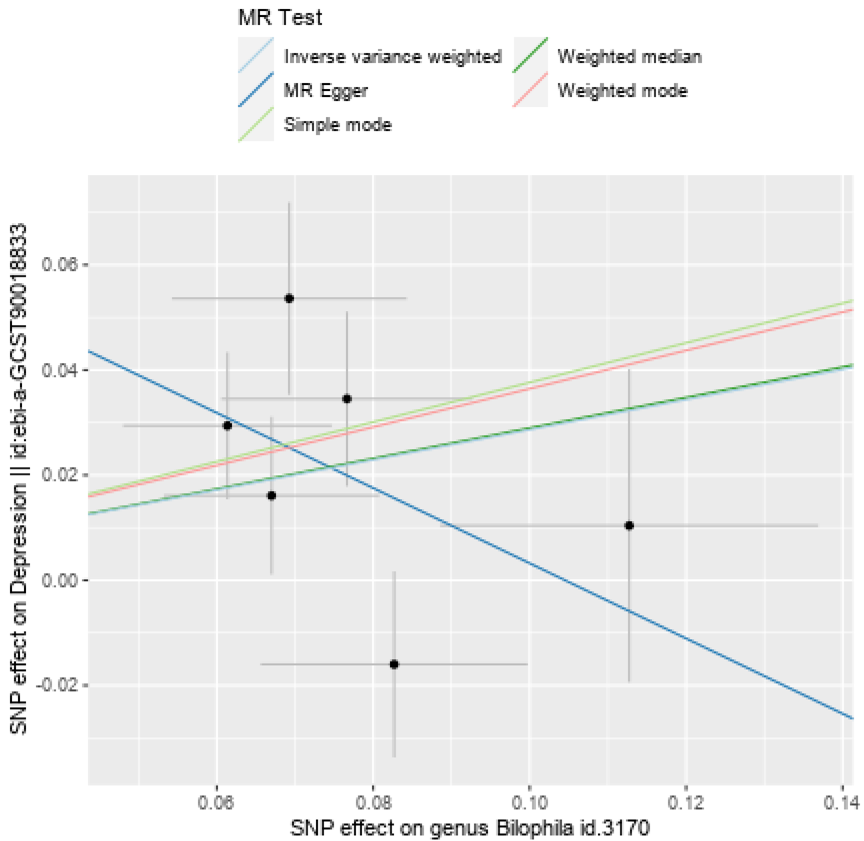
MR Test
Inverse variance weighted
MR Egger
Simple mode
Weighted median
Weighted mode
SNP effect on Depression || id:ebi-a-GCST90018833
0.06
0.04
0.02
0.00
-0.02
0.06
0.08
0.10
0.12
0.14
SNP effect on genus Bilophila id.3170

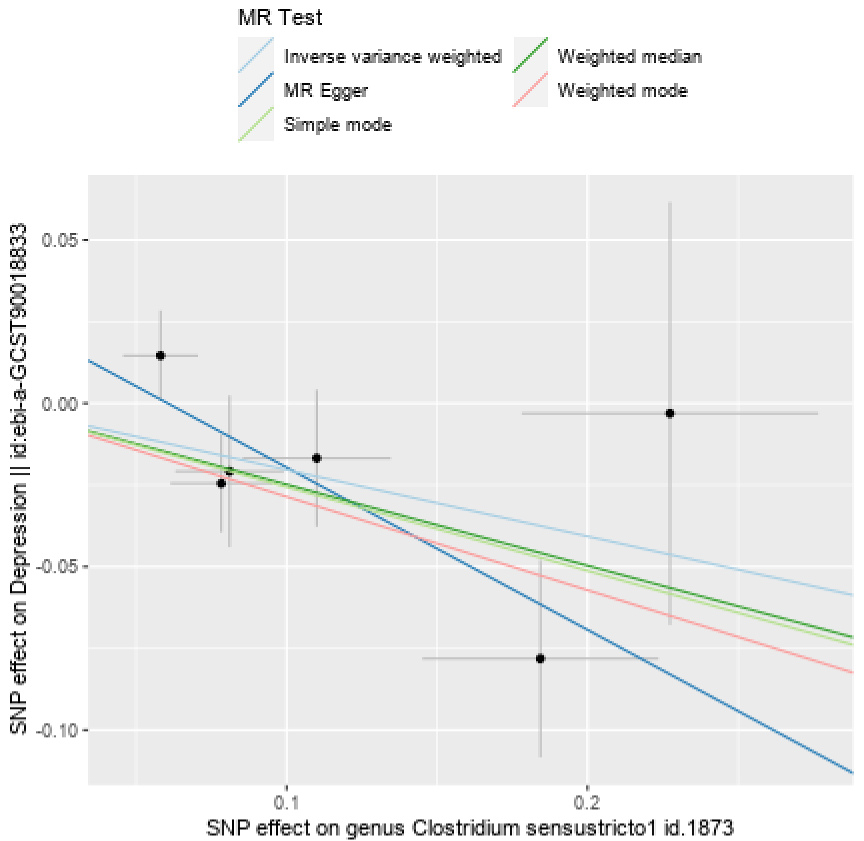

MR Test
Inverse variance weighted
MR Egger
Simple mode
Weighted median
Weighted mode
0.05
0.00
-0.05
-0.10
0.1
0.2
SNP effect on Depression || id:ebi-a-GCST90018833
SNP effect on genus Clostridium sensustricto1 id.1873

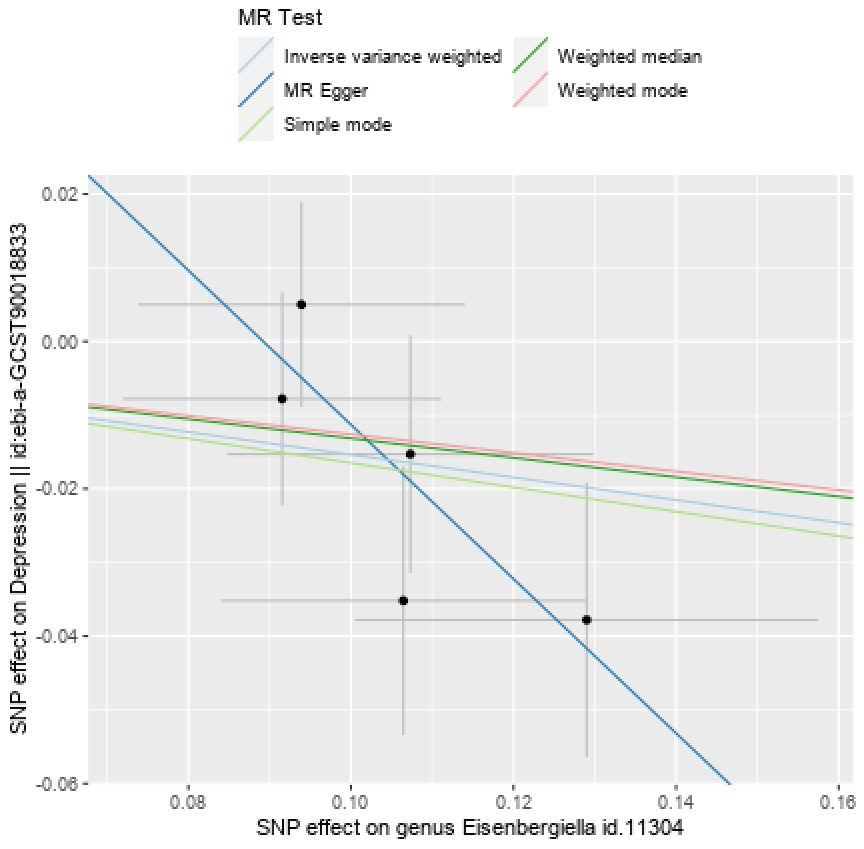
MR Test
Inverse variance weighted
MR Egger
Simple mode
Weighted median
Weighted mode
0.02
0.00
-0.02
-0.04
-0.06
SNP effect on Depression || id:ebi-a-GCST90018833
0.08
0.10
0.12
0.14
0.16
SNP effect on genus Eisenbergiella id.11304

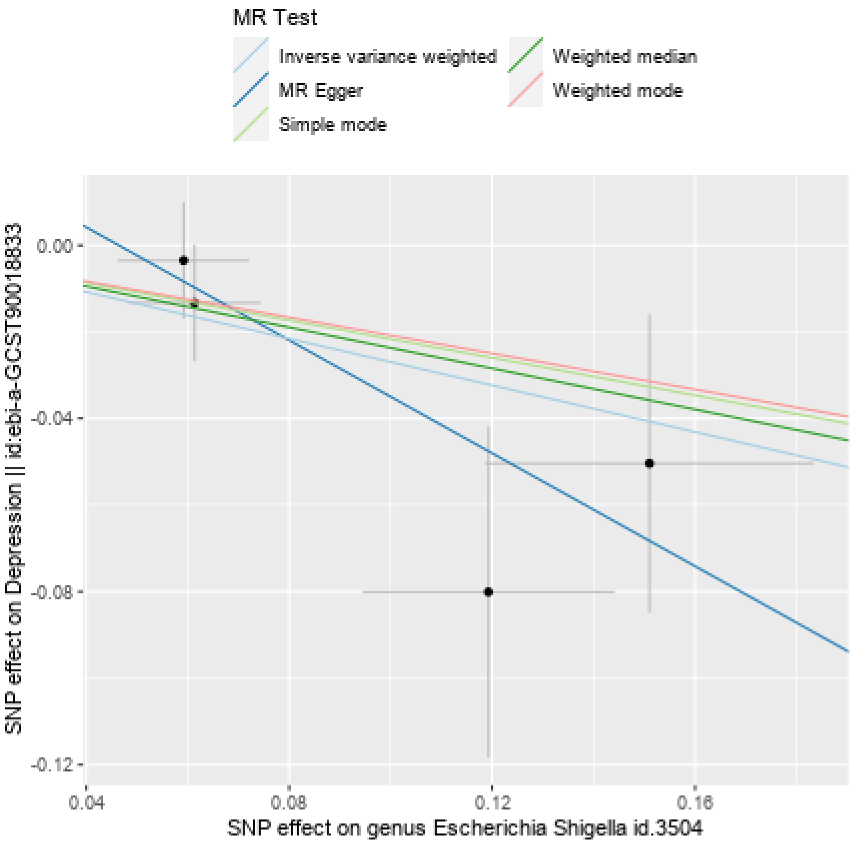
MR Test
Inverse variance weighted
MR Egger
Simple mode
Weighted median
Weighted mode
SNP effect on Depression || id:ebi-a-GCST90018833
0.00
-0.04
-0.08
-0.12
0.04
0.08
0.12
0.16
SNP effect on genus Escherichia Shigella id.3504

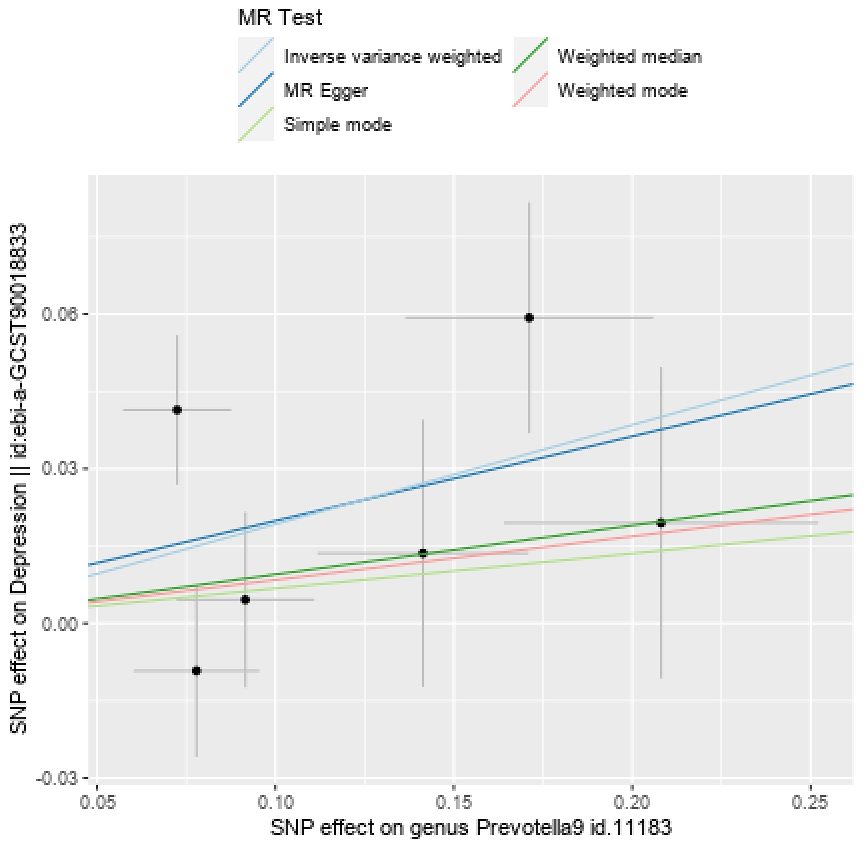
MR Test
Inverse variance weighted
MR Egger
Simple mode
Weighted median
Weighted mode
SNP effect on Depression || id:ebi-a-GCST90018833
0.06
0.03
0.00
-0.03
0.05
0.10
0.15
0.20
0.25
SNP effect on genus Prevotella9 id.11183
