# Supplementary material for: Causal relationships between gut microbiota and depression/anxiety disorders: A 2-sample Mendelian randomization study
Source: Medicine (Baltimore). 2024 Sep 6;103(36):e39543. doi: 10.1097/MD.0000000000039543 (PMC12431749; doi:10.1097/MD.0000000000039543)
Supplement: Supplementary file 2 [file medi-103-e39543-s002.pdf]

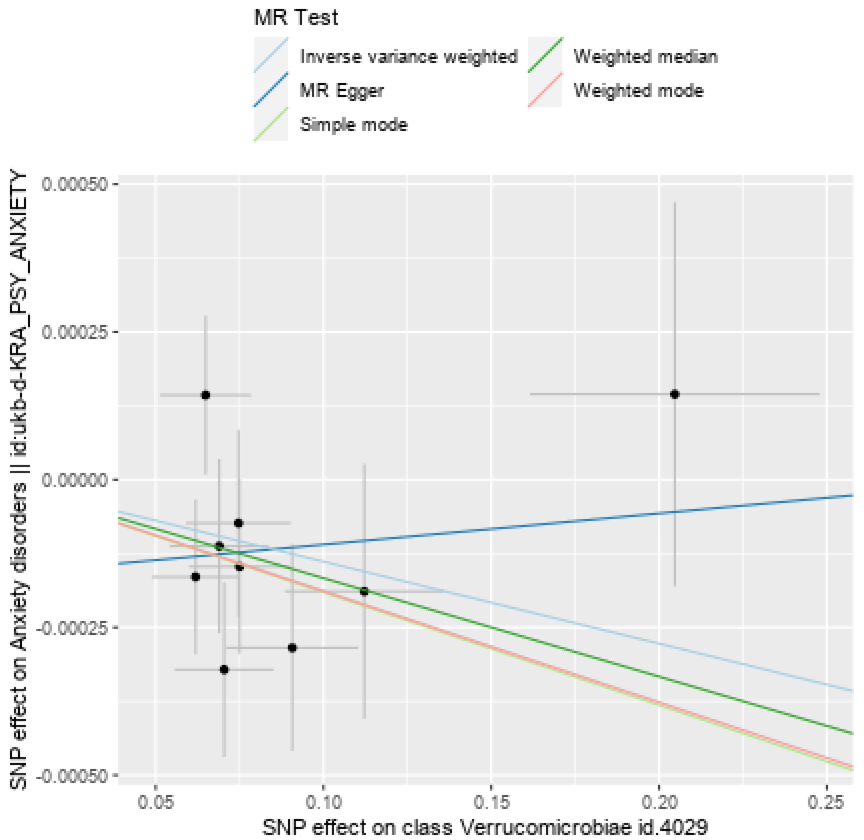

MR Test
Inverse variance weighted
MR Egger
Simple mode
Weighted median
Weighted mode
0.00050
0.00025
0.00000
-0.00025
-0.00050
SNP effect on Anxiety disorders || id:ukb-d-KRA_PSY_ANXIETY
0.05
0.10
0.15
0.20
0.25
SNP effect on class Verrucomicrobiae id.4029

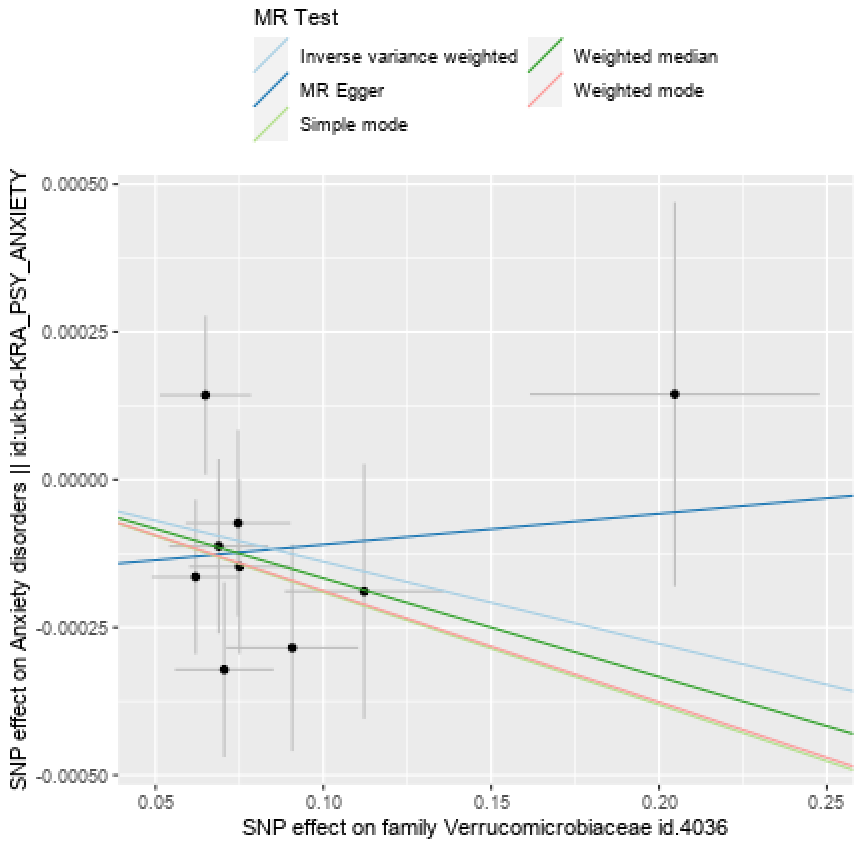
MR Test
Inverse variance weighted
MR Egger
Simple mode
Weighted median
Weighted mode
SNP effect on Anxiety disorders || id:ukb-d-KRA_PSY_ANXIETY
0.00050
0.00025
0.00000
-0.00025
-0.00050
0.05
0.10
0.15
0.20
0.25
SNP effect on family Verrucomicrobiaceae id.4036

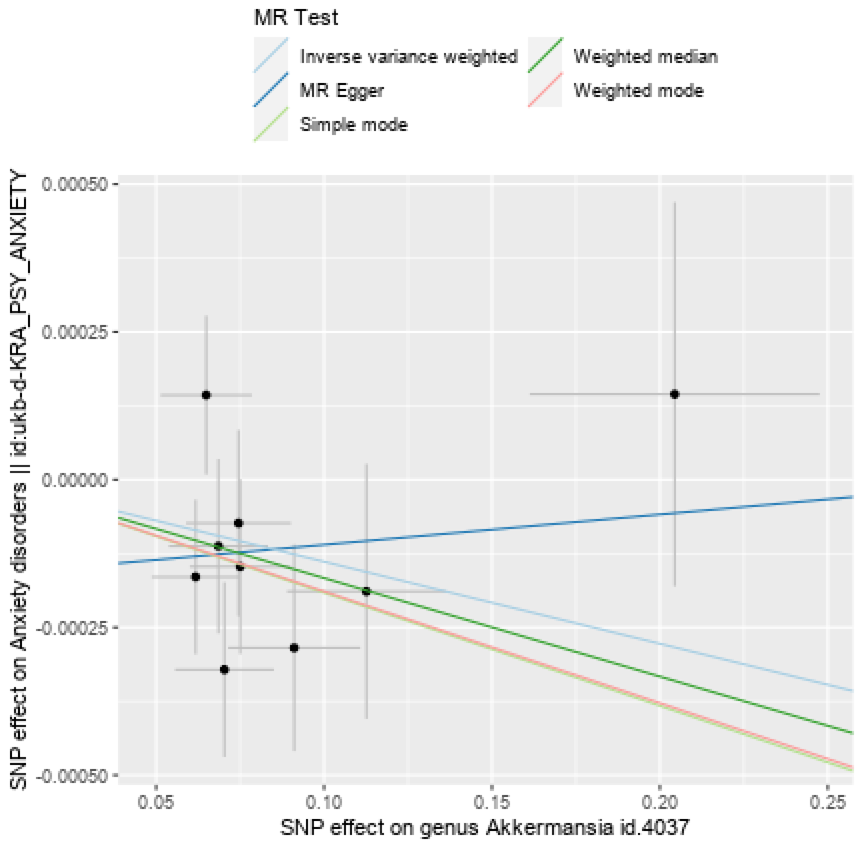
MR Test
Inverse variance weighted
MR Egger
Simple mode
Weighted median
Weighted mode
SNP effect on Anxiety disorders || id:ukb-d-KRA_PSY_ANXIETY
0.00050
0.00025
0.00000
-0.00025
-0.00050
0.05
0.10
0.15
0.20
0.25
SNP effect on genus Akkermansia id.4037

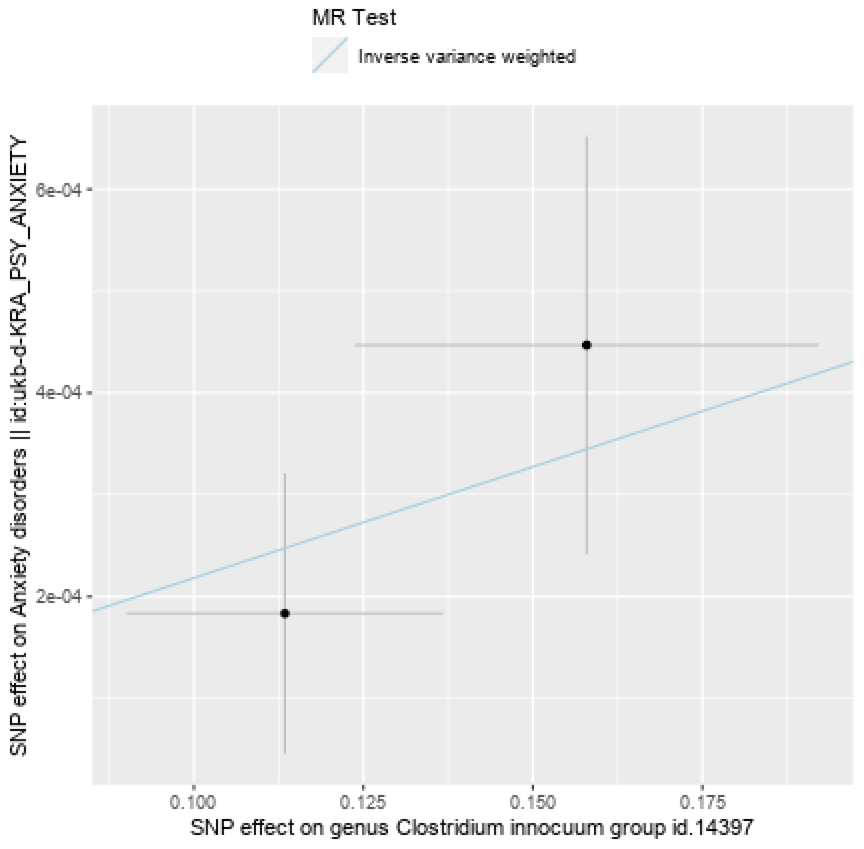
MR Test
Inverse variance weighted
SNP effect on Anxiety disorders || id:ukb-d-KRA_PSY_ANXIETY
6e-04
4e-04
2e-04
0.100
0.125
0.150
0.175
SNP effect on genus Clostridium innocuum group id.14397

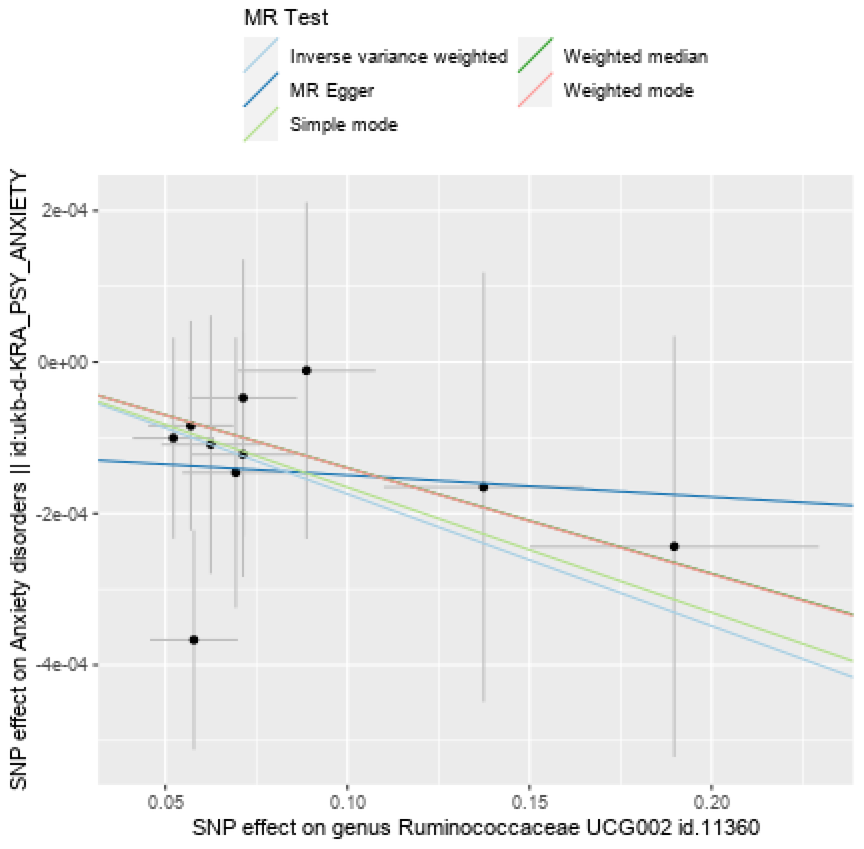

MR Test
Inverse variance weighted
MR Egger
Simple mode
Weighted median
Weighted mode
2e-04
0e+00
-2e-04
-4e-04
SNP effect on Anxiety disorders || id:ukb-d-KRA_PSY_ANXIETY
0.05
0.10
0.15
0.20
SNP effect on genus Ruminococcaceae UCG002 id.11360

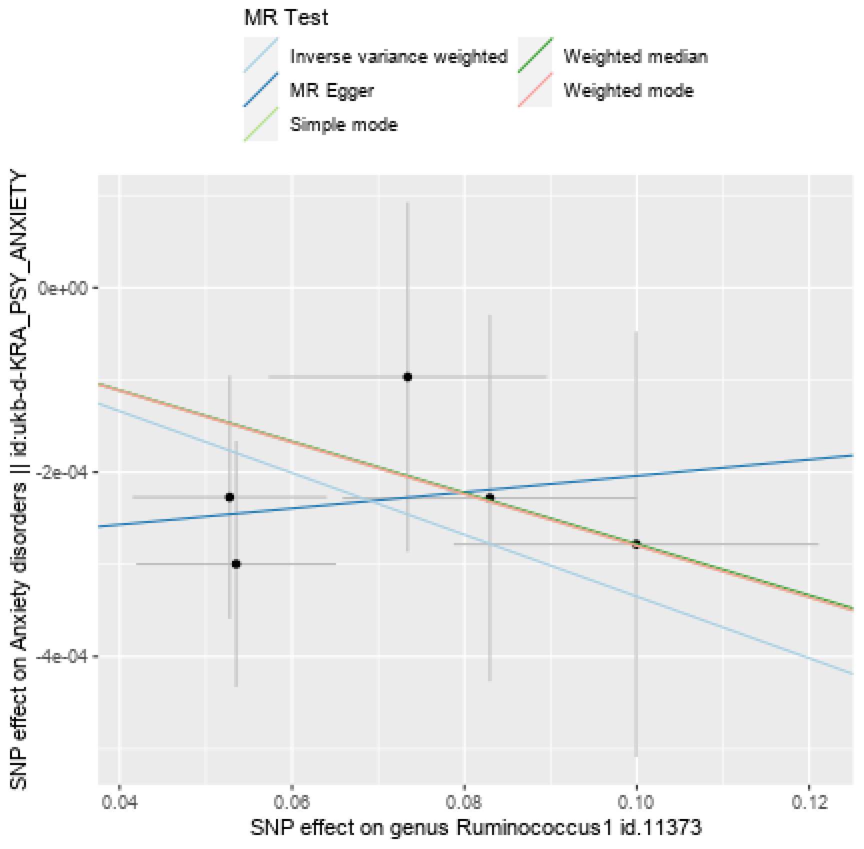

MR Test
Inverse variance weighted
MR Egger
Simple mode
Weighted median
Weighted mode
0e+00
-2e-04
-4e-04
SNP effect on Anxiety disorders || id:ukb-d-KRA_PSY_ANXIETY
0.04
0.06
0.08
0.10
0.12
SNP effect on genus Ruminococcus1 id.11373

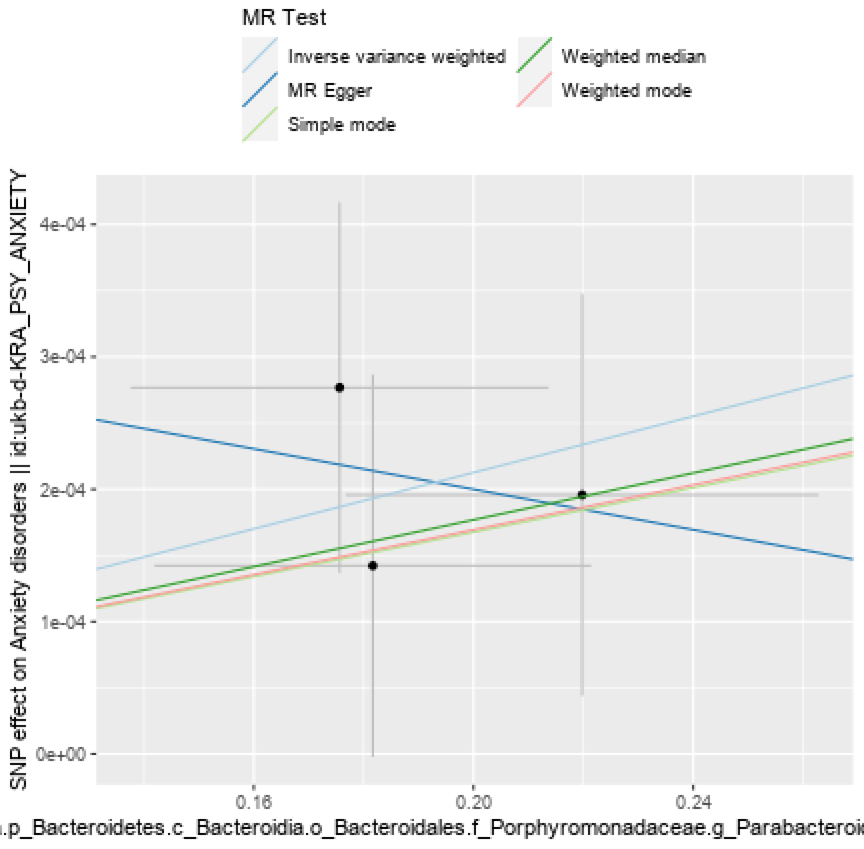
MR Test
Inverse variance weighted
MR Egger
Simple mode
Weighted median
Weighted mode
SNP effect on Anxiety disorders || id:ukb-d-KRA_PSY_ANXIETY
4e-04
3e-04
2e-04
1e-04
0e+00
0.16
0.20
0.24
.p_Bacteroidetes.c_Bacteroidia.o_Bacteroidales.f_Porphyromonadaceae.g_Parabacteroi

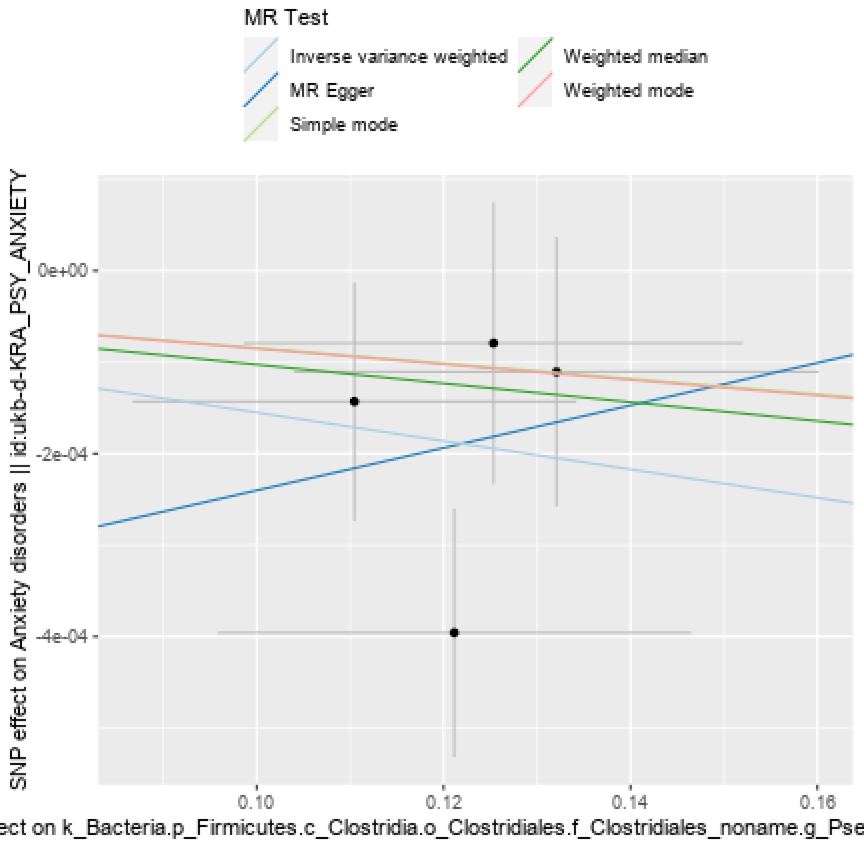

MR Test
Inverse variance weighted
MR Egger
Simple mode
Weighted median
Weighted mode
0e+00
-2e-04
-4e-04
SNP effect on Anxiety disorders || id:ukb-d-KRA_PSY_ANXIETY
0.10
0.12
0.14
0.16
ect on k_Bacteria.p_Firmicutes.c_Clostridia.o_Clostridiales.f_Clostridiales_noname.g_Pse

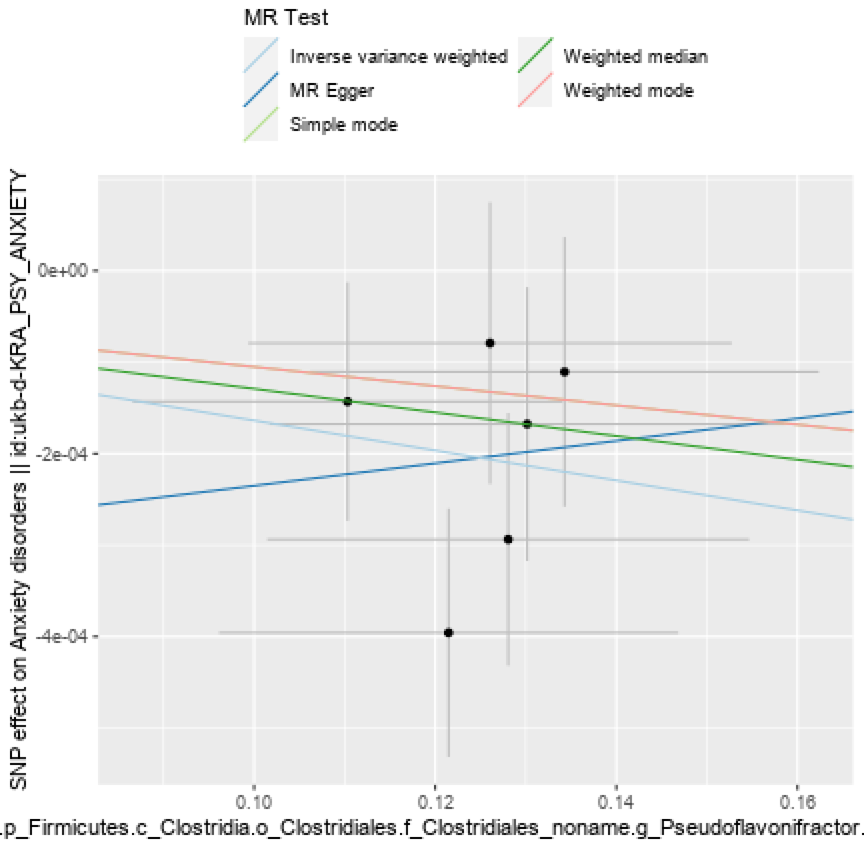
MR Test
Inverse variance weighted
MR Egger
Simple mode
Weighted median
Weighted mode
SNP effect on Anxiety disorders || id:ukb-d-KRA_PSY_ANXIETY
0e+00
-2e-04
-4e-04
0.10
0.12
0.14
0.16
.p_Firmicutes.c_Clostridia.o_Clostridiales.f_Clostridiales_noname.g_Pseudoflavonifractor.

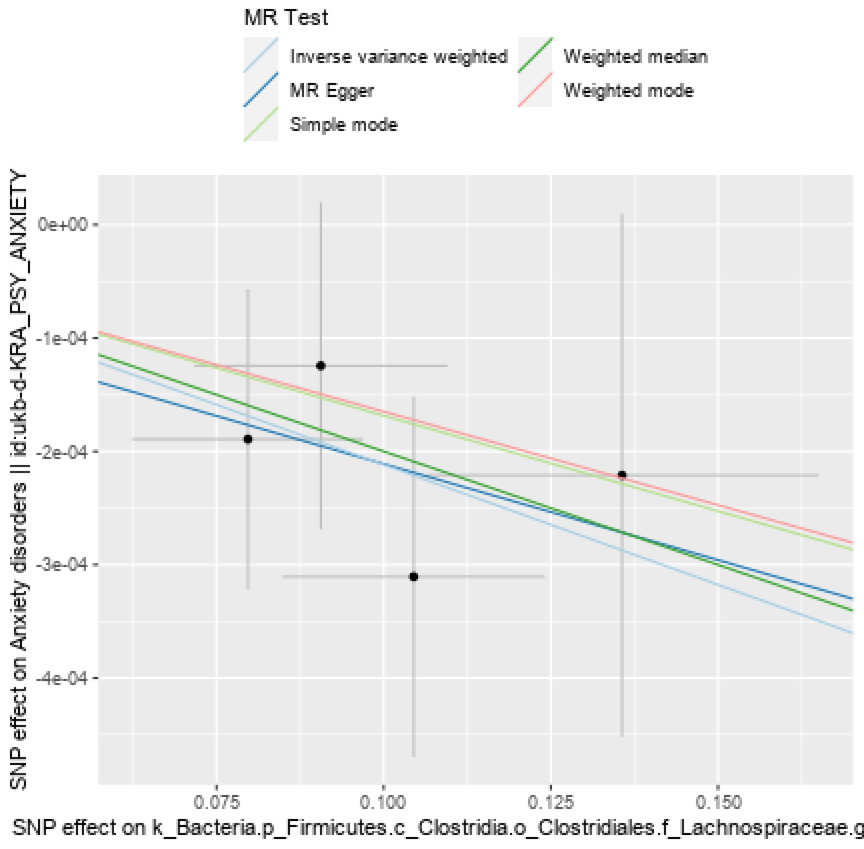
MR Test
Inverse variance weighted
MR Egger
Simple mode
Weighted median
Weighted mode
SNP effect on Anxiety disorders || id:ukb-d-KRA_PSY_ANXIETY
0e+00
-1e-04
-2e-04
-3e-04
-4e-04
0.075
0.100
0.125
0.150
SNP effect on k_Bacteria.p_Firmicutes.c_Clostridia.o_Clostridiales.f_Lachnospiraceae.g

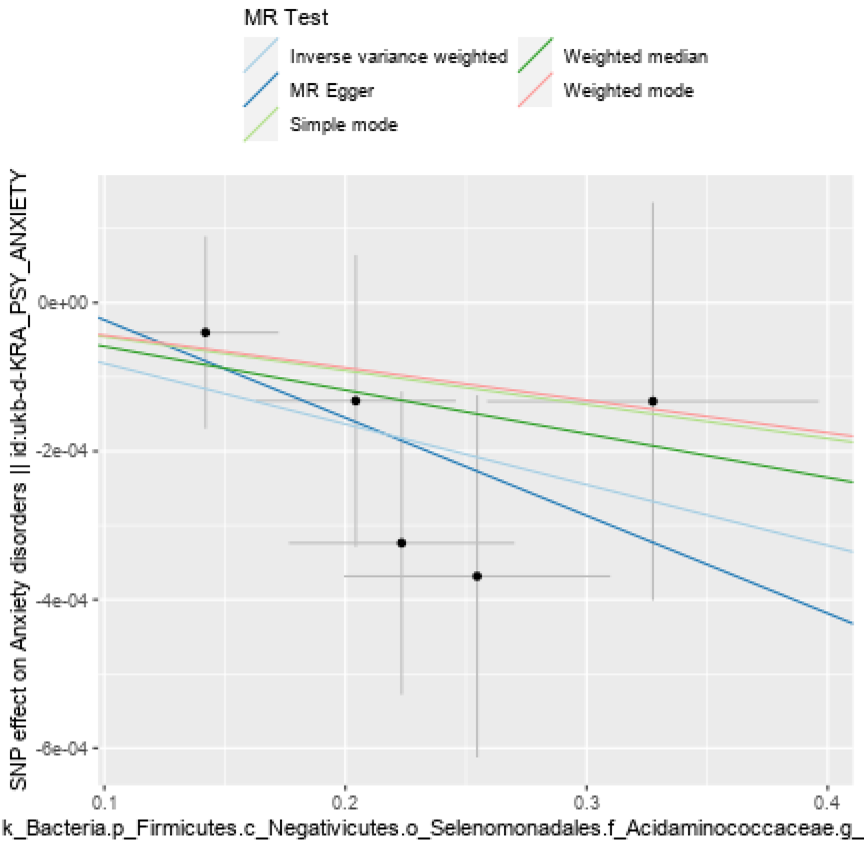

MR Test
Inverse variance weighted
MR Egger
Simple mode
Weighted median
Weighted mode
SNP effect on Anxiety disorders || id:ukb-d-KRA_PSY_ANXIETY
0e+00
-2e-04
-4e-04
-6e-04
0.1
0.2
0.3
0.4
k_Bacteria.p_Firmicutes.c_Negativicutes.o_Selenomonadales.f_Acidaminococcaceae.g_

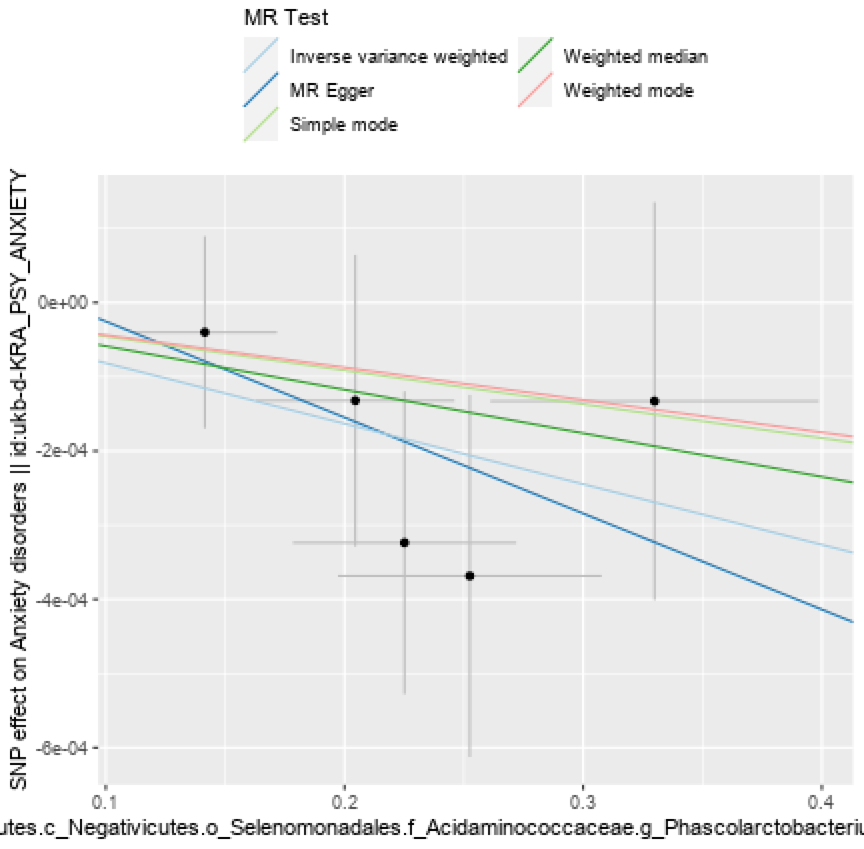

MR Test
Inverse variance weighted
MR Egger
Simple mode
Weighted median
Weighted mode
SNP effect on Anxiety disorders || id:ukb-d-KRA_PSY_ANXIETY
0e+00
-2e-04
-4e-04
-6e-04
0.1
0.2
0.3
0.4
utes.c_Negativicutes.o_Selenomonadales.f_Acidaminococcaceae.g_Phascolarctobacteriu

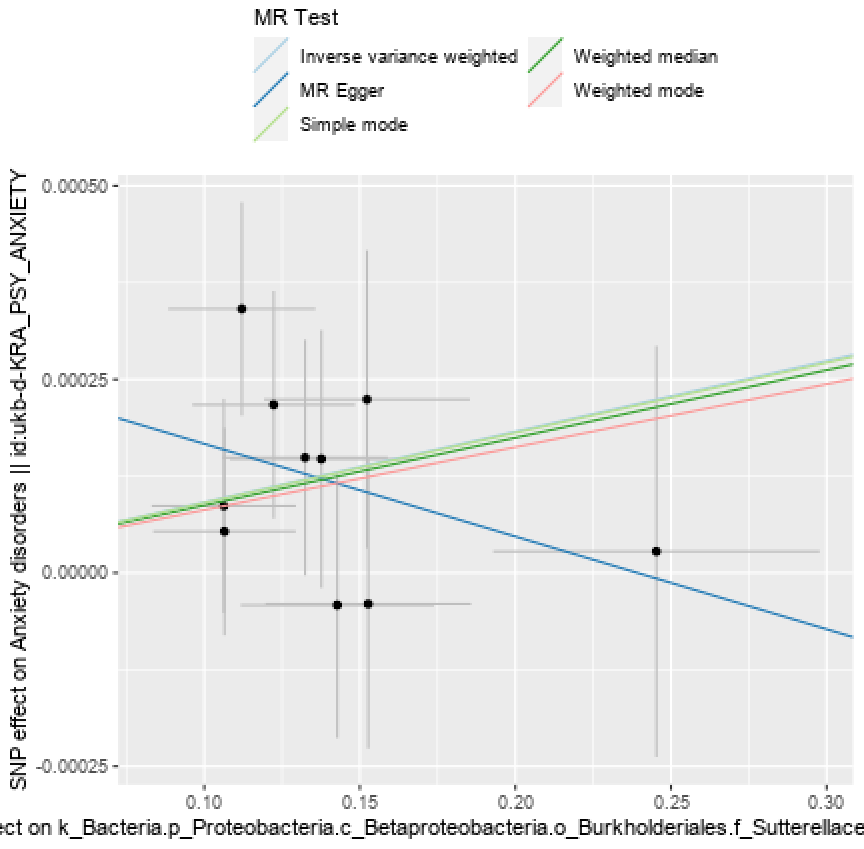

MR Test
Inverse variance weighted
MR Egger
Simple mode
Weighted median
Weighted mode
0.00050
0.00025
0.00000
-0.00025
SNP effect on Anxiety disorders || id:ukb-d-KRA_PSY_ANXIETY
0.10
0.15
0.20
0.25
0.30
ect on k_Bacteria.p_Proteobacteria.c_Betaproteobacteria.o_Burkholderiales.f_Sutterellace

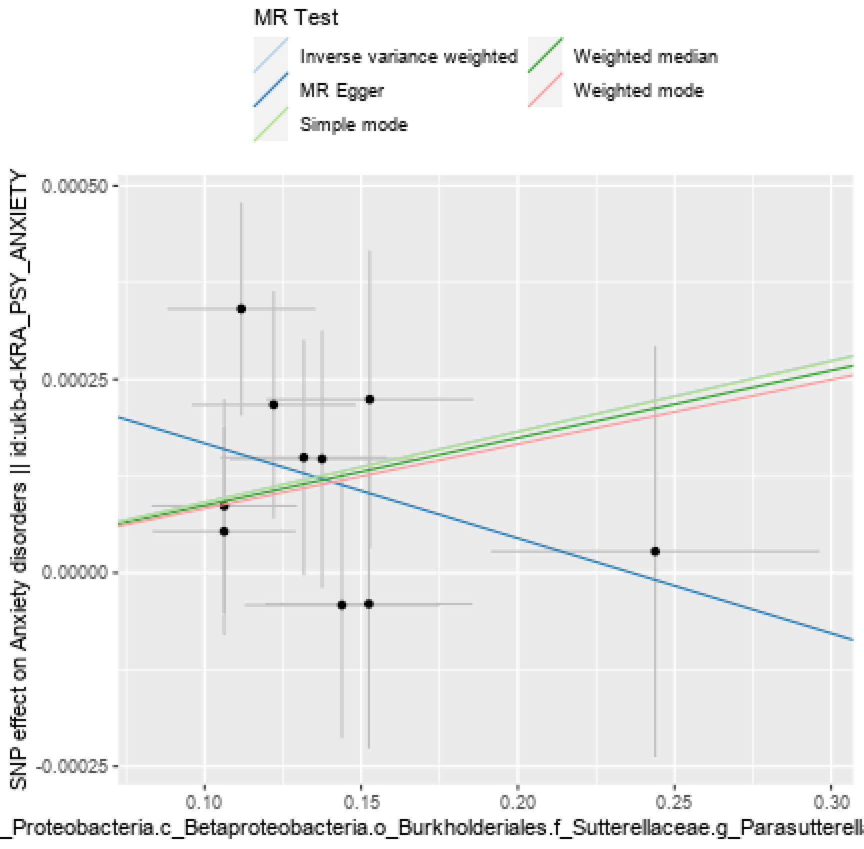
MR Test
Inverse variance weighted
MR Egger
Simple mode
Weighted median
Weighted mode
SNP effect on Anxiety disorders || id:ukb-d-KRA_PSY_ANXIETY
0.00050
0.00025
0.00000
-0.00025
0.10
0.15
0.20
0.25
0.30
_Proteobacteria.c_Betaproteobacteria.o_Burkholderiales.f_Sutterellaceae.g_Parasutterell

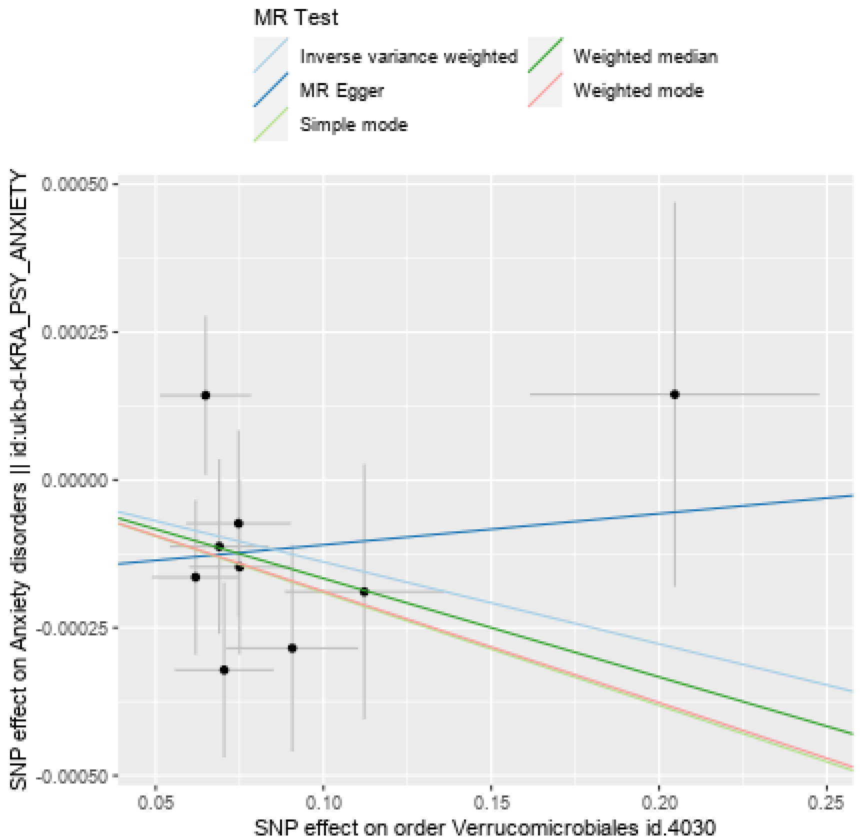
MR Test
Inverse variance weighted
MR Egger
Simple mode
Weighted median
Weighted mode
0.00050
0.00025
0.00000
-0.00025
-0.00050
0.05
0.10
0.15
0.20
0.25
SNP effect on Anxiety disorders || id:ukb-d-KRA_PSY_ANXIETY
SNP effect on order Verrucomicrobiales id.4030

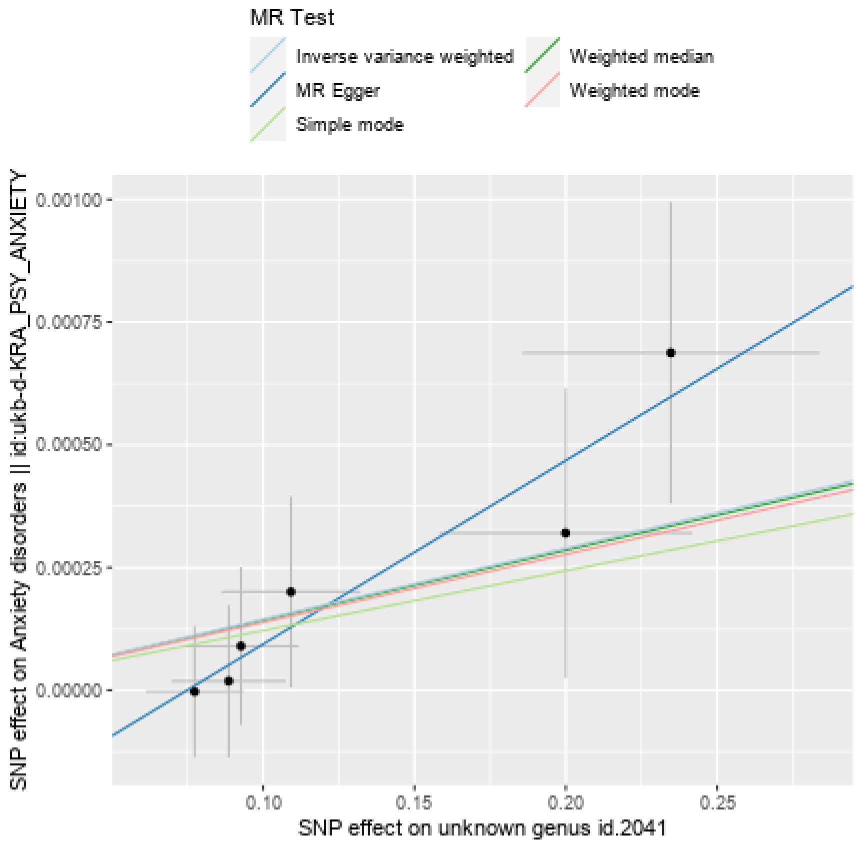
MR Test
Inverse variance weighted
MR Egger
Simple mode
Weighted median
Weighted mode
0.00100
0.00075
0.00050
0.00025
0.00000
0.10
0.15
0.20
0.25
SNP effect on Anxiety disorders || id:ukb-d-KRA_PSY_ANXIETY
SNP effect on unknown genus id.2041
