# Supplementary material for: Causal relationships between gut microbiota and depression/anxiety disorders: A 2-sample Mendelian randomization study
Source: Medicine (Baltimore). 2024 Sep 6;103(36):e39543. doi: 10.1097/MD.0000000000039543 (PMC12431749; doi:10.1097/MD.0000000000039543)
Supplement: Supplementary file 3 [file medi-103-e39543-s003.pdf]

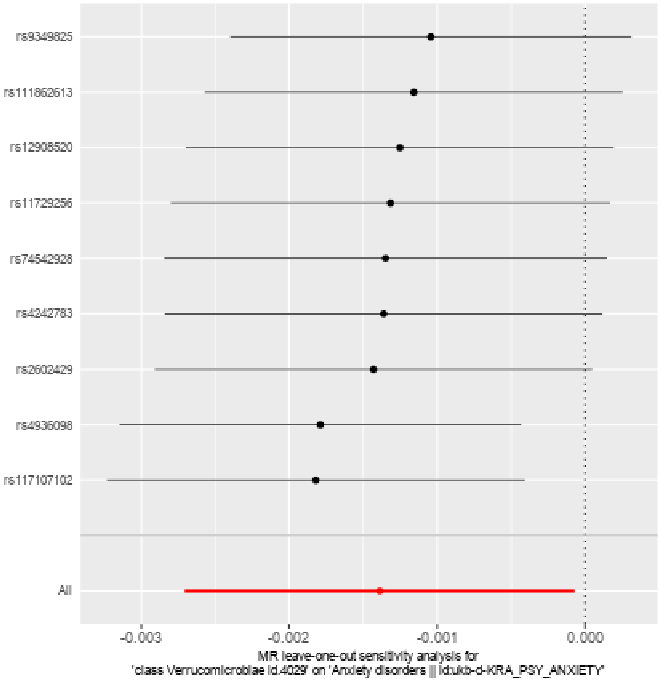
rs9349825
rs111862613
rs12908520
rs11729256
rs74542928
rs4242783
rs2602429
rs4936098
rs117107102
All
-0.003
-0.002
-0.001
0.000
MR leave-one-out sensitivity analysis for
'class Verrucomicrobiae id.4029' on 'Anxiety disorders || id:ukb-d-KRA_PSY_ANXIETY'

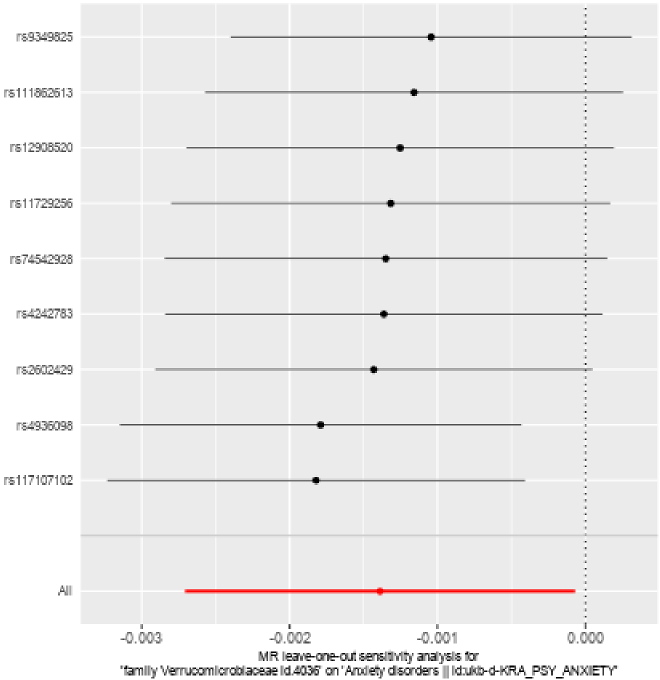

rs9349825
rs111862613
rs12908520
rs11729256
rs74542928
rs4242783
rs2602429
rs4936098
rs117107102
All
-0.003
-0.002
-0.001
0.000
MR leave-one-out sensitivity analysis for
'family Verrucomicrobiaceae id.4036' on 'Anxiety disorders || id:ukb-d-KRA_PSY_ANXIETY'

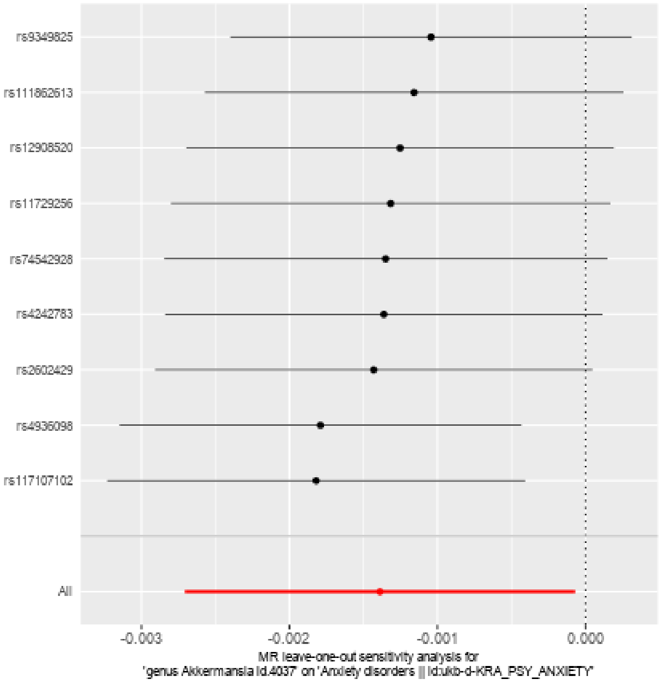
rs9349825
rs111862613
rs12908520
rs11729256
rs74542928
rs4242783
rs2602429
rs4936098
rs117107102
All
-0.003
-0.002
-0.001
0.000
MR leave-one-out sensitivity analysis for
'genus Akkermansia id.4037' on 'Anxiety disorders || id:ukb-d-KRA_PSY_ANXIETY'

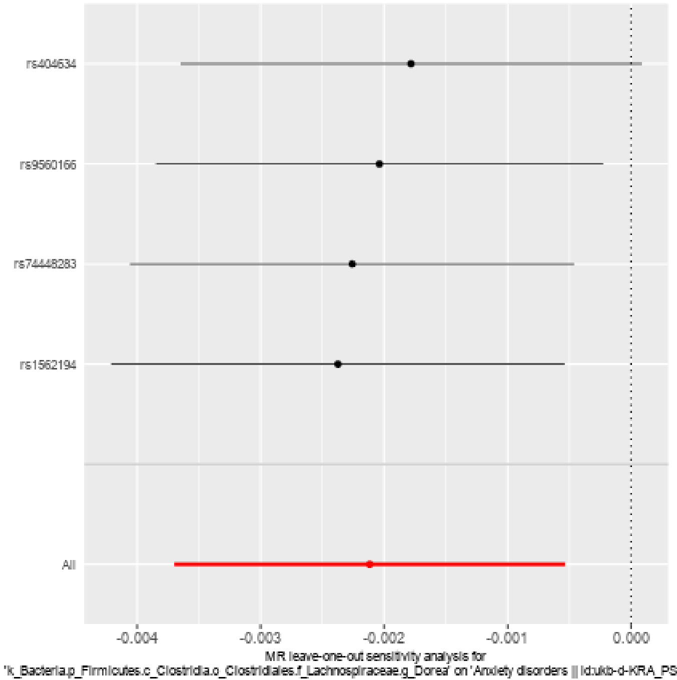
rs404634
rs9560166
rs74448283
rs1562194
All
-0.004
-0.003
-0.002
-0.001
0.000
MR leave-one-out sensitivity analysis for
'k_Bacteria.p_Firmicutes.c_Clostridia.o_Clostridiales.f_Lachnospiraceae.g_Dorea' on 'Anxiety disorders || id:ukb-d-KRA_PS

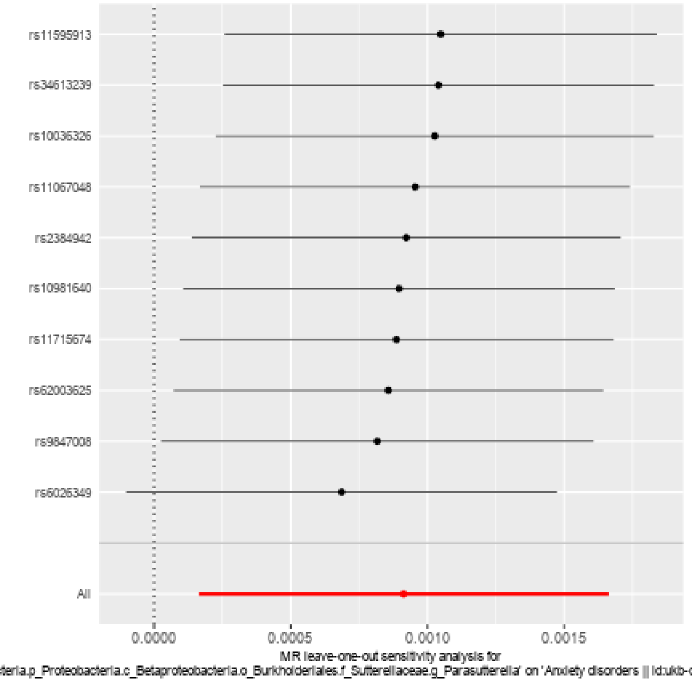

rs11595913
rs34613239
rs10036326
rs11067048
rs2384942
rs10981640
rs11715674
rs62003625
rs9847008
rs6026349
All
0.0000
0.0005
0.0010
0.0015
MR leave-one-out sensitivity analysis for
teria.p_Proteobacteria.c_Betaproteobacteria.o_Burkholderiales.f_Sutterellaceae.g_Parasutterella' on 'Anxiety disorders || id:ukb-c

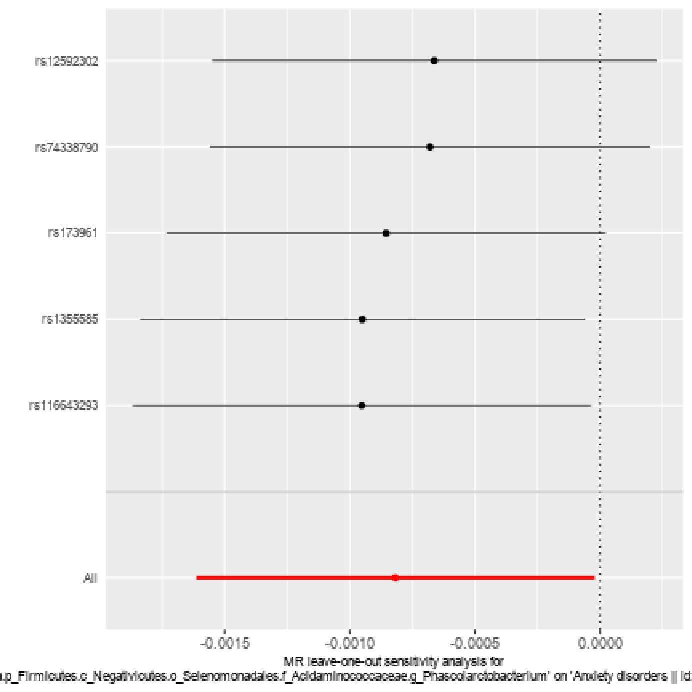
rs12592302
rs74338790
rs173961
rs1355585
rs116643293
All
-0.0015
-0.0010
-0.0005
0.0000
MR leave-one-out sensitivity analysis for
.p_Firmicutes.c_Negativicutes.o_Selenomonadales.f_Acidaminococcaceae.g_Phascolarctobacterium' on 'Anxiety disorders || id:

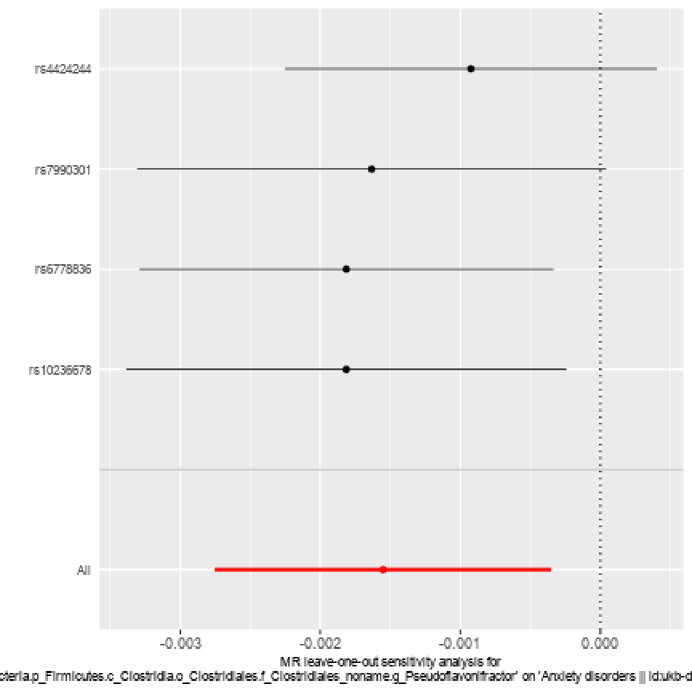

rs4424244
rs7990301
rs6778836
rs10236678
All
-0.003
-0.002
-0.001
0.000
MR leave-one-out sensitivity analysis for
cteria.p_Firmicutes.c_Clostridia.o_Clostridiales.f_Clostridiales_noname.g_Pseudoflavonifractor' on 'Anxiety disorders || id:ukb-d

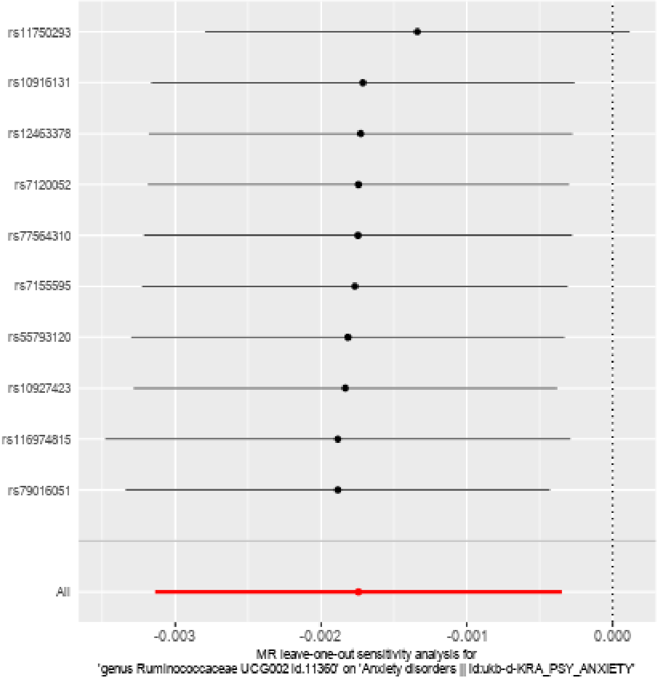
rs11750293
rs10916131
rs12463378
rs7120052
rs77564310
rs7155595
rs55793120
rs10927423
rs116974815
rs79016051
All
-0.003
-0.002
-0.001
0.000
MR leave-one-out sensitivity analysis for
'genus Ruminococcaceae UCG002 id.11360' on 'Anxiety disorders || id:ukb-d-KRA_PSY_ANXIETY'

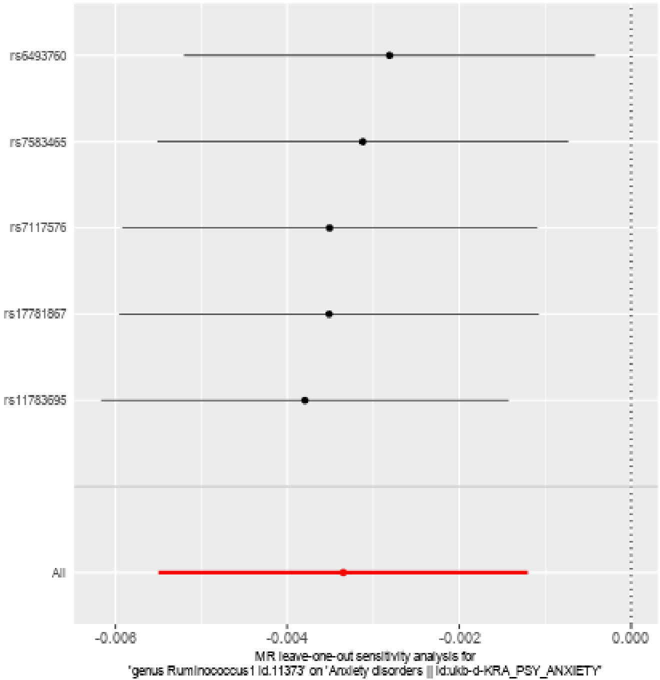
rs6493760
rs7583465
rs7117576
rs17781867
rs11783695
All
-0.006
-0.004
-0.002
0.000
MR leave-one-out sensitivity analysis for
'genus Ruminococcus1 id.11373' on 'Anxiety disorders || id:ukb-d-KRA_PSY_ANXIETY'

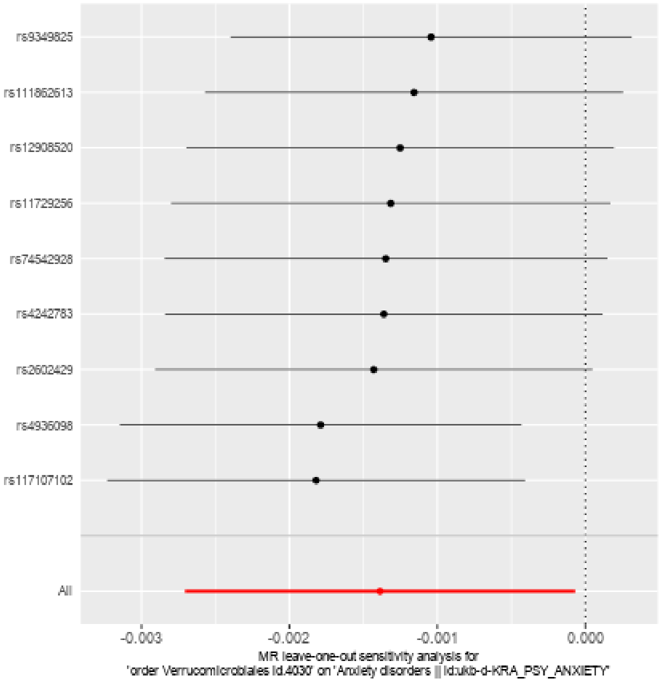
rs9349825
rs111862613
rs12908520
rs11729256
rs74542928
rs4242783
rs2602429
rs4936098
rs117107102
All
-0.003
-0.002
-0.001
0.000
MR leave-one-out sensitivity analysis for
'order Verrucomicrobiales id.4030' on 'Anxiety disorders || id:ukb-d-KRA_PSY_ANXIETY'

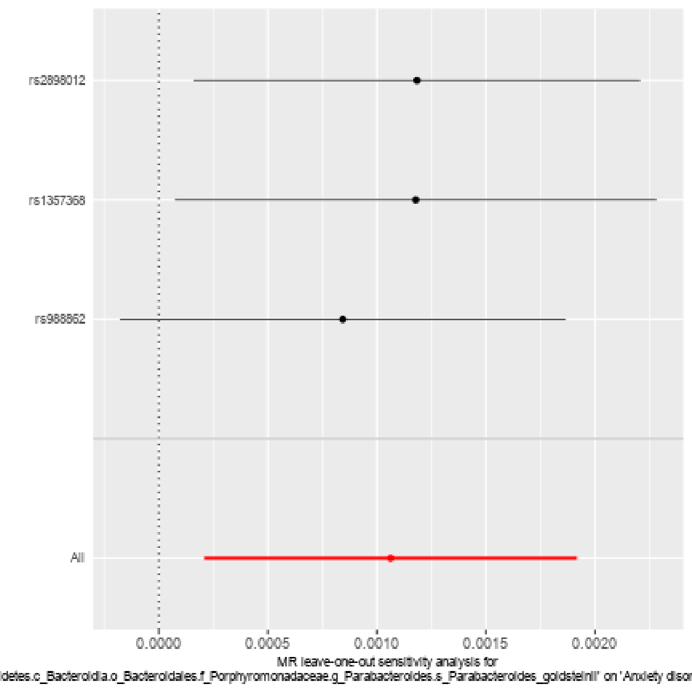

rs2898012
rs1357368
rs988862
All
0.0000
0.0005
0.0010
0.0015
0.0020
MR leave-one-out sensitivity analysis for
idetes.c_Bacteroidia.o_Bacteroidales.f_Porphyromonadaceae.g_Parabacteroides.s_Parabacteroides_goldsteinii' on 'Anxiety disor

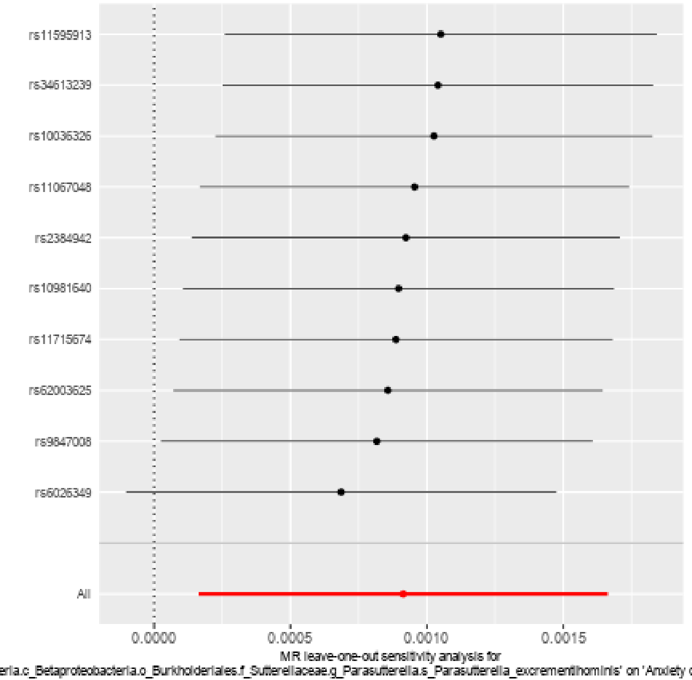
rs11595913
rs34613239
rs10036326
rs11067048
rs2384942
rs10981640
rs11715674
rs62003625
rs9847008
rs6026349
All
0.0000
0.0005
0.0010
0.0015
MR leave-one-out sensitivity analysis for
erla.c_Betaproteobacteria.o_Burkholderiales.f_Sutterellaceae.g_Parasutterella.s_Parasutterella_excrementihominis' on 'Anxiety d

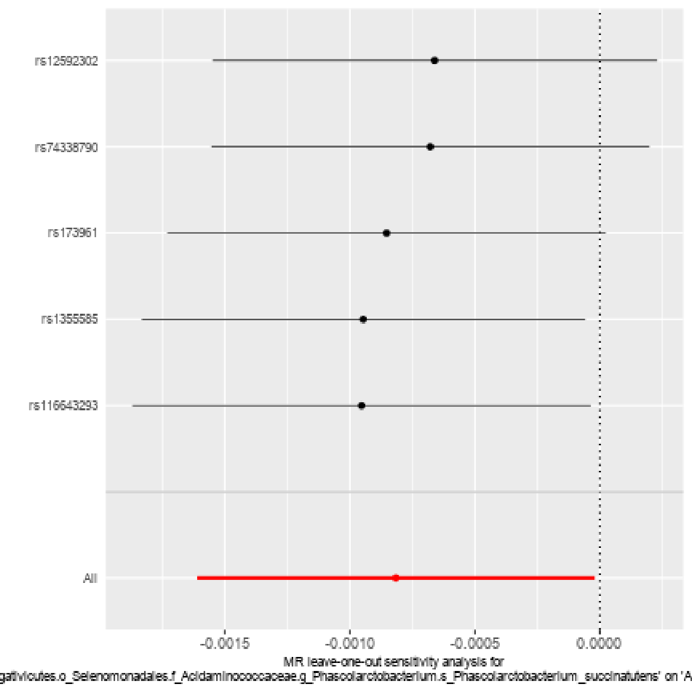

rs12592302
rs74338790
rs173961
rs1355585
rs116643293
All
-0.0015
-0.0010
-0.0005
0.0000
MR leave-one-out sensitivity analysis for
gativicutes.o_Selenomonadales.f_Acidaminococcaceae.g_Phascolarctobacterium.s_Phascolarctobacterium_succinatutens' on 'A

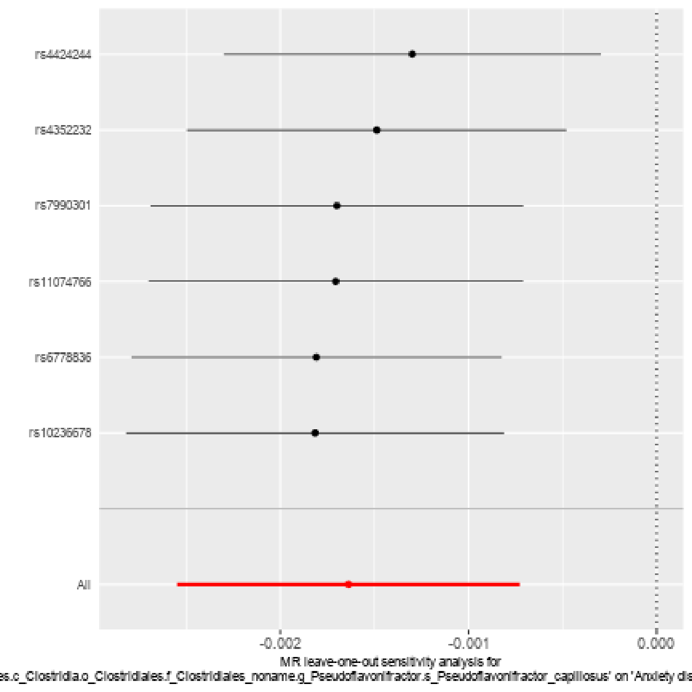

rs4424244
rs4352232
rs7990301
rs11074766
rs6778836
rs10236678
All
-0.002
-0.001
0.000
MR leave-one-out sensitivity analysis for
es.c_Clostridia.o_Clostridiales.f_Clostridiales_noname.g_Pseudoflavonifractor.s_Pseudoflavonifractor_capillosus' on 'Anxiety dis

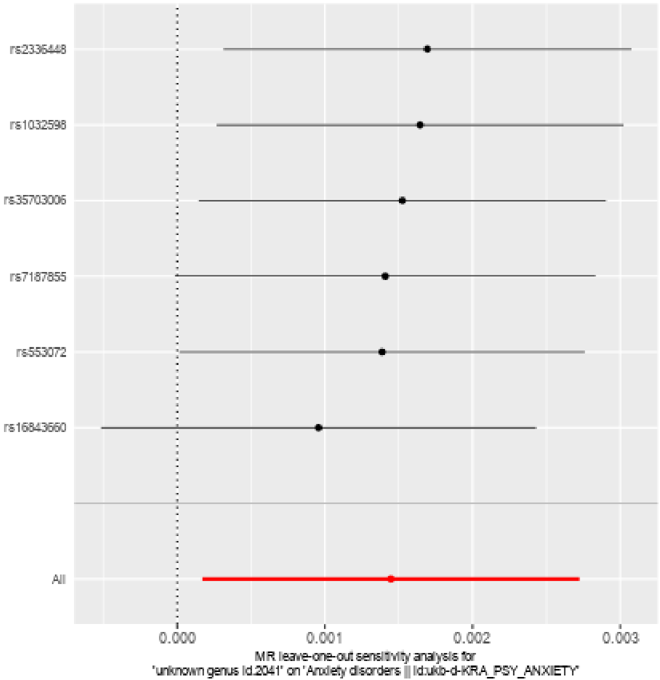
rs2336448
rs1032598
rs35703006
rs7187855
rs553072
rs16843660
All
0.000
0.001
0.002
0.003
MR leave-one-out sensitivity analysis for
'unknown genus id.2041' on 'Anxiety disorders || id:ukb-d-KRA_PSY_ANXIETY'

Supplementary Figure 1. Leave-one-out analysis of anxiety disorder.
