# Supplementary material for: Causal relationships between gut microbiota and depression/anxiety disorders: A 2-sample Mendelian randomization study
Source: Medicine (Baltimore). 2024 Sep 6;103(36):e39543. doi: 10.1097/MD.0000000000039543 (PMC12431749; doi:10.1097/MD.0000000000039543)
Supplement: Supplementary file 4 [file medi-103-e39543-s004.pdf]

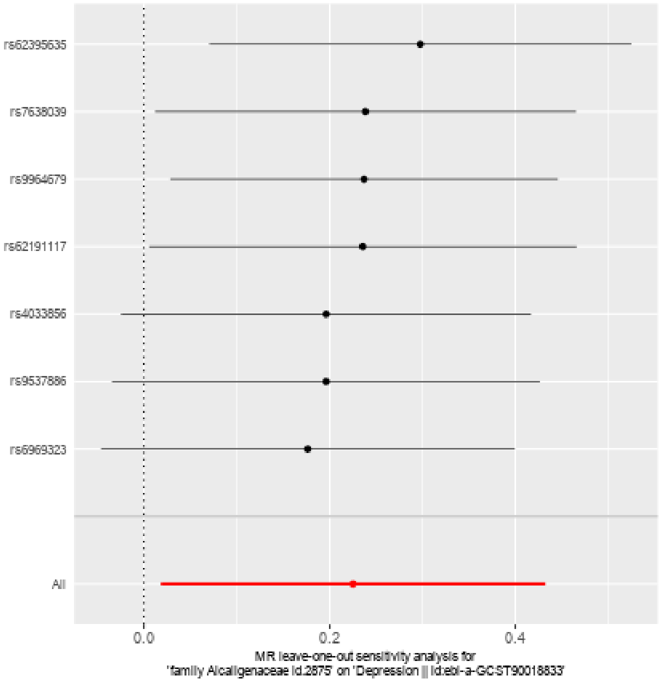
rs62395635
rs7638039
rs9964679
rs62191117
rs4033856
rs9537886
rs6969323
All
0.0
0.2
0.4
MR leave-one-out sensitivity analysis for
'family Alcaligenaceae id.2875' on 'Depression || id:ebi-a-GCST90018833'

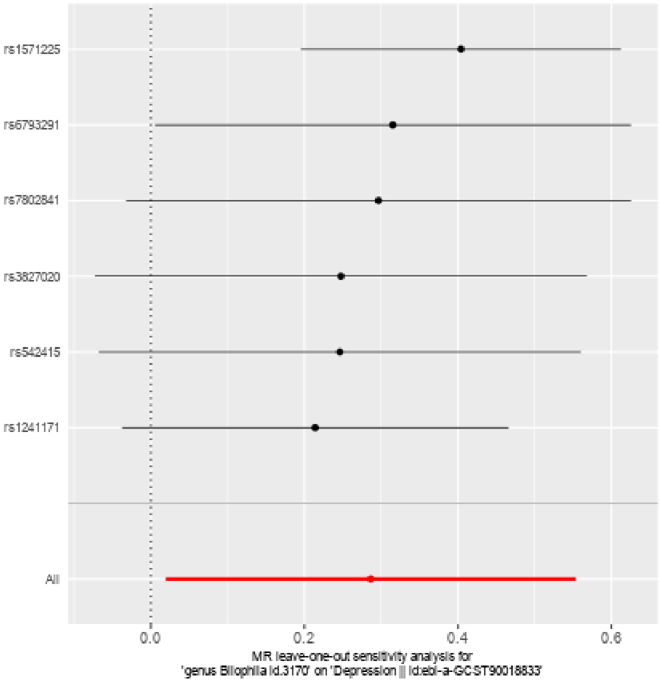
rs1571225
rs6793291
rs7802841
rs3827020
rs542415
rs1241171
All
0.0
0.2
0.4
0.6
MR leave-one-out sensitivity analysis for
'genus Bilophila id.3170' on 'Depression || id:ebi-a-GCST90018833'

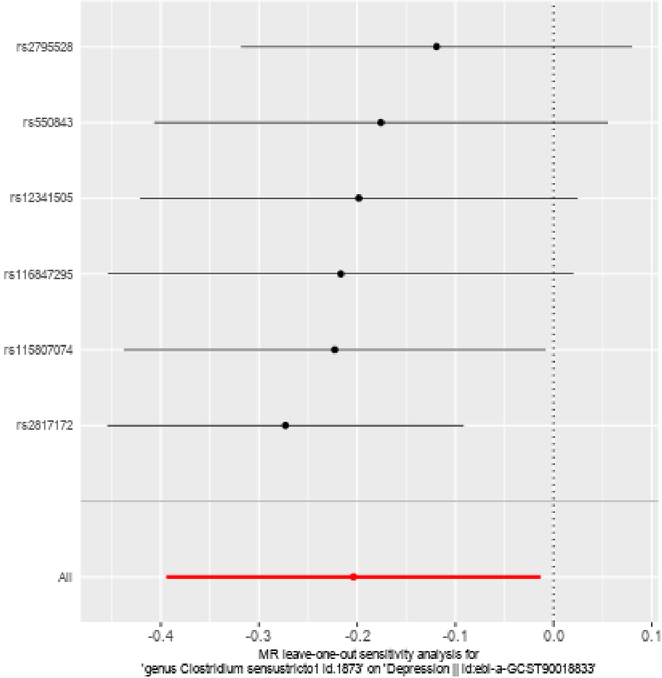

rs2795528
rs550843
rs12341505
rs116847295
rs115807074
rs2817172
All
-0.4
-0.3
-0.2
-0.1
0.0
0.1
MR leave-one-out sensitivity analysis for
'genus Clostridium sensustricto1 id.1873' on 'Depression || id:ebi-a-GCST90018833'

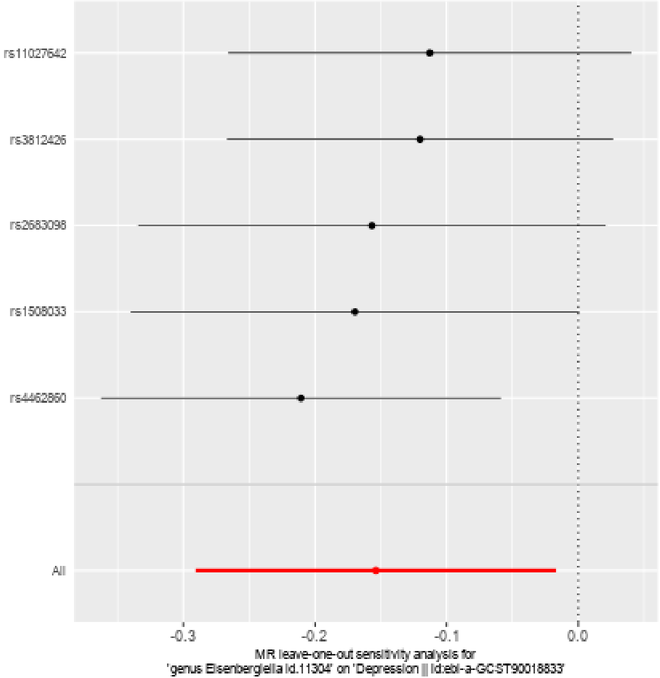
rs11027642
rs3812426
rs2683098
rs1508033
rs4462860
All
-0.3
-0.2
-0.1
0.0
MR leave-one-out sensitivity analysis for
'genus Eisenbergiella id.11304' on 'Depression || id:ebi-a-GCST90018833'

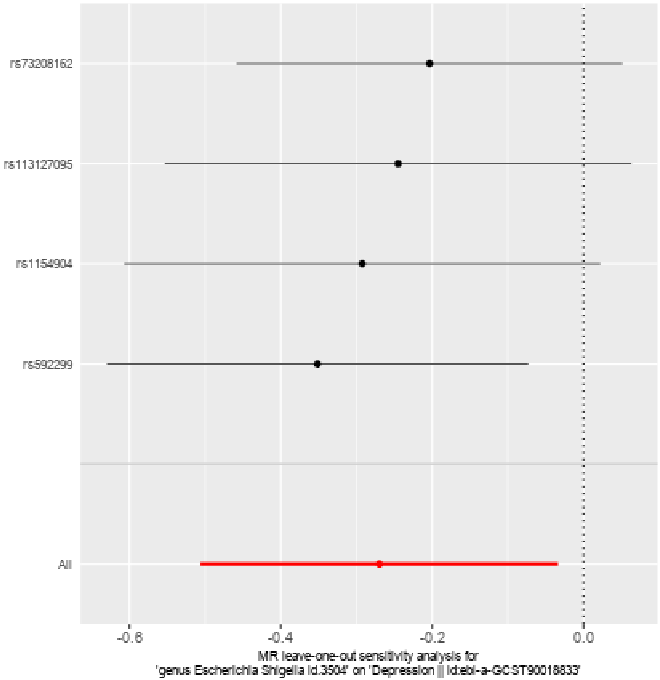

rs73208162
rs113127095
rs1154904
rs592299
All
-0.6
-0.4
-0.2
0.0
MR leave-one-out sensitivity analysis for
'genus Escherichia Shigella id.3504' on 'Depression || id:ebi-a-GCST90018833'

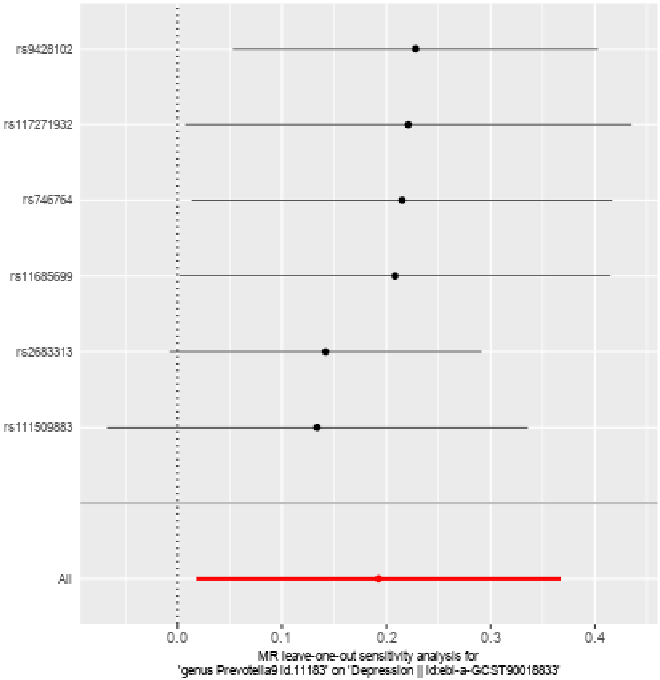

rs9428102
rs117271932
rs746764
rs11685699
rs2683313
rs111509883
All
0.0
0.1
0.2
0.3
0.4
MR leave-one-out sensitivity analysis for
'genus Prevotella9 id.11183' on 'Depression || id:ebi-a-GCST90018833'

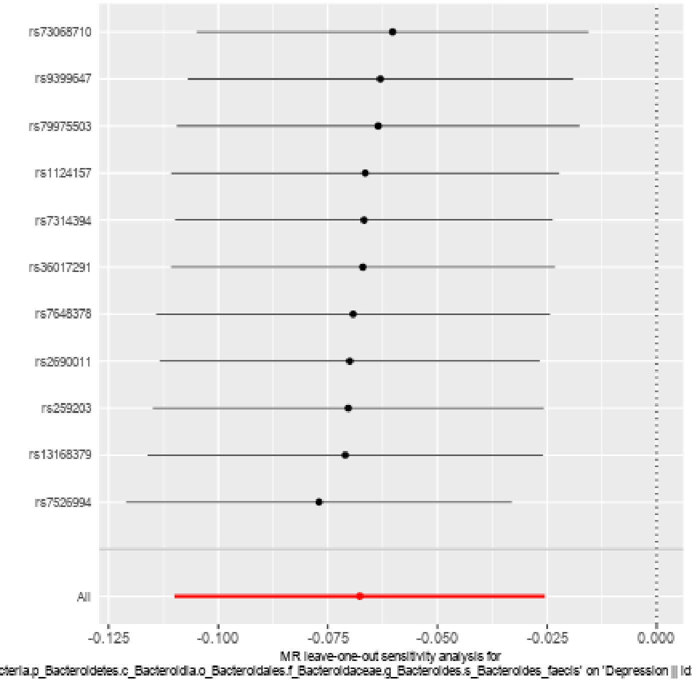

rs73068710
rs9399647
rs79975503
rs1124157
rs7314394
rs36017291
rs7648378
rs2690011
rs259203
rs13168379
rs7526994
All
-0.125
-0.100
-0.075
-0.050
-0.025
0.000
MR leave-one-out sensitivity analysis for
cteria.p_Bacteroidetes.c_Bacteroidia.o_Bacteroidales.f_Bacteroidaceae.g_Bacteroides.s_Bacteroides_faecis' on 'Depression || Id

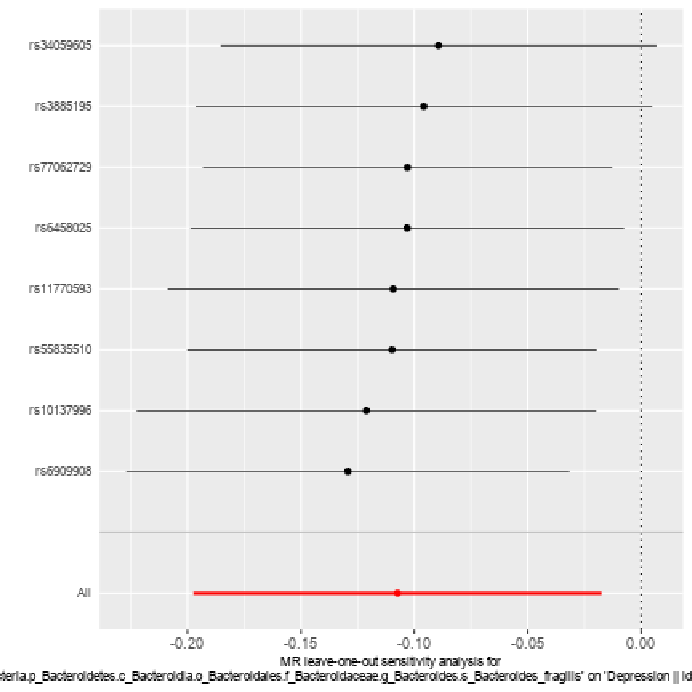

rs34059605
rs3885195
rs77062729
rs6458025
rs11770593
rs55835510
rs10137996
rs6909908
All
-0.20
-0.15
-0.10
-0.05
0.00
MR leave-one-out sensitivity analysis for
cteria.p_Bacteroidetes.c_Bacteroidia.o_Bacteroidales.f_Bacteroidaceae.g_Bacteroides.s_Bacteroides_fragilis' on 'Depression || id

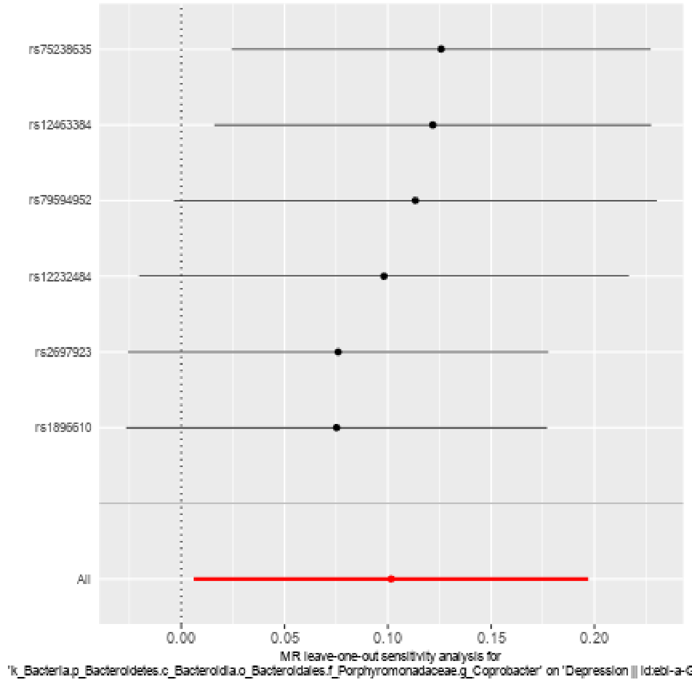
rs75238635
rs12463384
rs79594952
rs12232484
rs2697923
rs1896610
All
0.00
0.05
0.10
0.15
0.20
MR leave-one-out sensitivity analysis for
'k_Bacteria.p_Bacteroidetes.c_Bacteroidia.o_Bacteroidales.f_Porphyromonadaceae.g_Coprobacter' on 'Depression || id:ebi-a-G

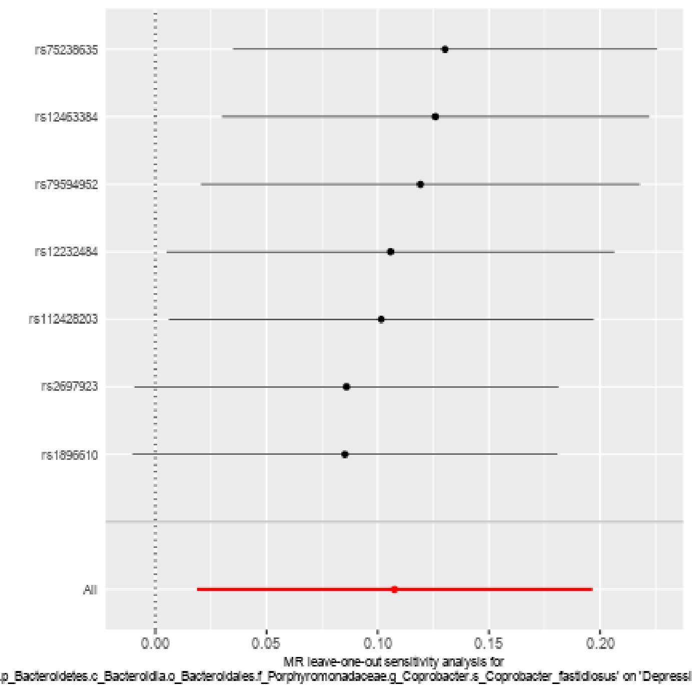

rs75238635
rs12463384
rs79594952
rs12232484
rs112428203
rs2697923
rs1896610
All
0.00
0.05
0.10
0.15
0.20
MR leave-one-out sensitivity analysis for
p_Bacteroidetes.c_Bacteroidia.o_Bacteroidales.f_Porphyromonadaceae.g_Coprobacter.s_Coprobacter_fastidiosus' on 'Depressi

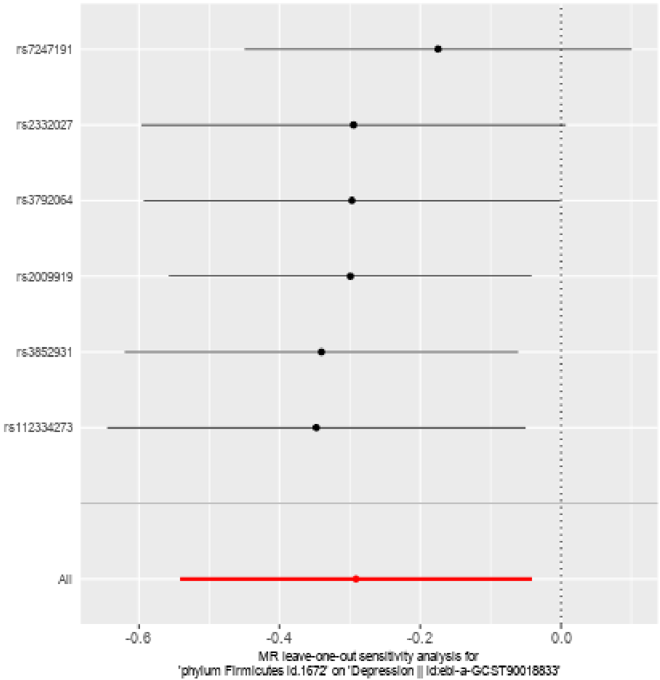
rs7247191
rs2332027
rs3792064
rs2009919
rs3852931
rs112334273
All
-0.6
-0.4
-0.2
0.0
MR leave-one-out sensitivity analysis for
'phylum Firmicutes id.1672' on 'Depression || id:ebi-a-GCST90018833'

Supplementary Figure 2. Leave-one-out analysis of depression.
